# Charge separation and carrier dynamics in donor-acceptor heterojunction photovoltaic systems

**DOI:** 10.1063/1.4996409

**Published:** 2017-12-19

**Authors:** Joël Teuscher, Jan C. Brauer, Andrey Stepanov, Alicia Solano, Ariadni Boziki, Majed Chergui, Jean-Pierre Wolf, Ursula Rothlisberger, Natalie Banerji, Jacques-E. Moser

**Affiliations:** 1 Photochemical Dynamics Group, Institute of Chemical Sciences and Engineering, École Polytechnique Fédérale de Lausanne, 1015 Lausanne, Switzerland; 2 Lausanne Centre for Ultrafast Science (LACUS), École Polytechnique Fédérale de Lausanne, 1015 Lausanne, Switzerland; 3 FemtoMat Group, Department of Chemistry, Université de Fribourg, 1700 Fribourg, Switzerland; 4 GAP-Biophotonics Group, Department of Applied Physics, Université de Genève, 1205 Geneva, Switzerland; 5 Laboratory of Computational Chemistry and Biochemistry, Institute of Chemical Sciences and Engineering, École Polytechnique Fédérale de Lausanne, 1015 Lausanne, Switzerland; 6 Laboratory of Ultrafast Spectroscopy, Institute of Chemical Sciences and Engineering, École Polytechnique Fédérale de Lausanne, 1015 Lausanne, Switzerland

## Abstract

Electron transfer and subsequent charge separation across donor-acceptor heterojunctions remain the most important areas of study in the field of third-generation photovoltaics. In this context, it is particularly important to unravel the dynamics of individual ultrafast processes (such as photoinduced electron transfer, carrier trapping and association, and energy transfer and relaxation), which prevail in materials and at their interfaces. In the frame of the National Center of Competence in Research “Molecular Ultrafast Science and Technology,” a research instrument of the Swiss National Science Foundation, several groups active in the field of ultrafast science in Switzerland have applied a number of complementary experimental techniques and computational simulation tools to scrutinize these critical photophysical phenomena. Structural, electronic, and transport properties of the materials and the detailed mechanisms of photoinduced charge separation in dye-sensitized solar cells, conjugated polymer- and small molecule-based organic photovoltaics, and high-efficiency lead halide perovskite solar energy converters have been scrutinized. Results yielded more than thirty research articles, an overview of which is provided here.

## I. INTRODUCTION AND SCOPE

Dye-sensitized mesoscopic solar cells (DSSCs), organic photovoltaic devices (OPV), and emerging hybrid organic-inorganic lead halide perovskite cells (PSCs) belong to a new generation of photovoltaic solar energy converters based on cheap, solution-processable materials. Contrary to the first-generation Si and GaAs solar cells, these systems separate the functions of light absorption and carrier transport. Light harvesting is carried out by an active material, in which photon absorption generates a local, generally short-lived charge separation. Charge transfer (CT) across specific contacts with a donor material able to transport positive carriers, on the one side, and an acceptor material constituting an electron transmitting medium, on the other side, prevents the charge recombination and allows the build-up of a significant photovoltage across the device. These types of solar cells do not depend on a built-in electric field at a *p-n* junction, but rather rely on prompt electron transfer (ET) at interfaces between materials, which energy levels are closely aligned. They are, therefore, commonly referred to as donor-acceptor heterojunction (DAH) photovoltaic systems.

Generation, thermalisation, trapping, interfacial transfer, and recombination of photoexcited charge carriers, as well as the dynamics of excitonic species, often occur on femtosecond to picosecond timescales. In the frame of the National Center of Competence in Research NCCR-MUST (Molecular Ultrafast Science and Technology), a research instrument of the Swiss National Science Foundation, five research groups active in the field of ultrafast science in Switzerland have combined a number of complementary experimental techniques and computational simulation tools to scrutinize these critical photophysical phenomena. The detailed understanding of ultrafast carrier dynamics brought by experimental measurements and calculations is not only important on a fundamental point of view, but it also provides the essential feedback to the design and selection of materials, morphology, heterostructures, and interfaces that enable improved photovoltaic performance.

The present review summarizes the results of this common endeavor, during which established femtosecond laser techniques, such as transient absorption (TAS), time-resolved terahertz (TRTS), and time-resolved photoemission spectroscopies were used, while additional experimental tools, such as time-resolved electroabsorption (TREAS) and picosecond X-ray absorption spectroscopies, for instance, needed to be developed specifically.

## II. PHOTOINDUCED CHARGE SEPARATION AT DONOR-ACCEPTOR HETEROJUNCTIONS

**FIG. 1.**
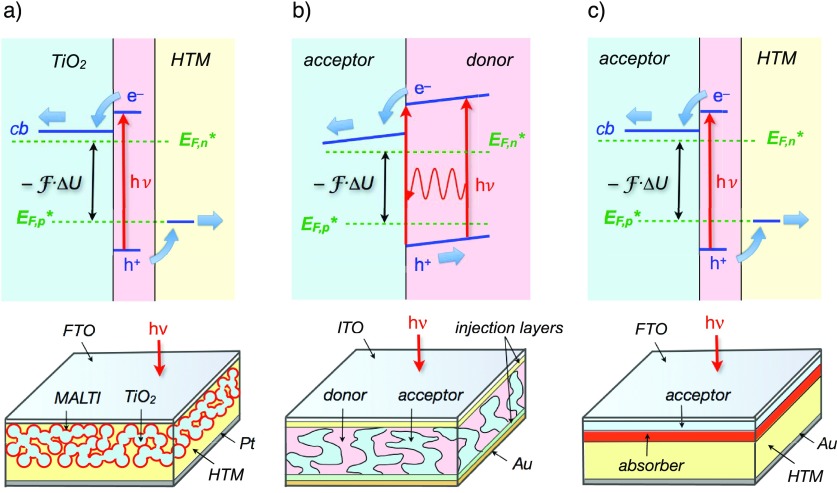
Energetic scheme and typical architecture of various types of photovoltaic systems based on donor-acceptor heterojunctions: (a) Dye-sensitized solar cells (DSSCs), (b) Polymer OPV bulk heterojunction cells, and (c) Planar small-molecule-based OPV and perovskite solar cells. The voltage across the device Δ*U* is given by the energy difference separating the quasi Fermi levels for electrons (E*_F,n_) and that for holes (E*_F,p_): –F·Δ*U*, where F is the Faraday constant.

Figure 1 presents in a schematic way the various photovoltaic systems that have been considered. Mesoscopic dye-sensitized solar cells [DSSCs, Fig. 1(a)] are constituted by a monolayer of a molecular dye adsorbed on nanocrystalline titanium dioxide particles sintered together as to form a highly porous, continuous framework.^1^ The latter acts as the acceptor and the electron-transporting medium. The donor can be either a liquid electrolyte containing a redox couple or a solid-state organic hole-transport material (HTM).^2^ The functioning principle of this type of photovoltaic system is based on the kinetic competition between various electron transfer processes.^2–4^ The initial charge separation, in particular, requires that electron injection from the dye's photoexcited state into the conduction band (CB) of TiO_2_ occurs before radiative and non-radiative deactivation or reductive quenching by the donor take place. The electronic excited states of dye molecules possessing heavy atoms and, therefore, experiencing a strong spin-orbit coupling, have extended lifetimes that can reach tens to hundreds of nanoseconds in the case of Ru(II) complexes. The lifetime of the singlet excited state of efficient organic dye sensitizers, however, can be as short as 100 ps. This implies that interfacial electron transfer with a time constant ≤1 ps is typically required in this case to compete against deactivation pathways and ensure an injection quantum yield close to unity.

Charge injection into the continuum of acceptor levels constituted by the conduction band of the semiconductor is intriguing by several aspects. The time constant for photoinduced interfacial electron transfer (ET) was found to vary from a few femtoseconds to hundreds of picoseconds, depending on the sensitizer molecule.^4^ Charge transfer times ≤25 fs indicate that the reaction takes place on a time scale that is comparable to or even shorter than that of nuclear motion associated with high-frequency intramolecular vibrations. In this case, the injection process cannot be described within the current Marcus-Levich-Jortner-Gerischer theory, which postulates vibration-mediated ET at thermal equilibrium.^4,6,7^ The notion that the electron is transferred to the solid well before the vibrational relaxation of the photoexcited sensitizer leads to the questions of the distribution of the excitation excess energy between hot carriers and hot dye oxidized states and the stabilization of the charge separated state. Thermalisation and trapping of conduction band electrons and the formation of interfacial charge transfer excitons (CTE) have in particular been addressed during the project.

Dye-sensitizer molecules do not only need to inject promptly into the conduction band of TiO_2_, they must also encompass a number of other important structural properties, allowing them in particular to impair fast charge recombination between injected electrons and positive charges carried by the oxidized states of the dye and the hole transporting medium.^2^ Structural design has been guided by computational molecular calculations and experimental ultrafast monitoring of the excited state dynamics.

Organic semiconductors consist of carbon-based, π-conjugated molecules or polymers, which can be solution-processed or vacuum-deposited in order to obtain thin films for optoelectronic applications, such as organic solar cells. Because of their low dielectric constant, organic solids are excitonic in nature, meaning that the electrons and holes in the excited state are strongly bound, unlike for inorganic materials, where free charges can be directly generated by light absorption.

Plastic photovoltaic devices usually rely on a conjugated polymer acting simultaneously as the active absorber and the hole-transporting material [Fig. 1(b)]. Upon photoexcitation, localized (Frenkel) excitons are generated that need to diffuse to the interface with an acceptor material—typically the fullerene soluble derivative phenyl-C_61_-butyric acid methyl ester (PCBM)—and dissociate before they recombine. Electrons injected in the acceptor material are thus separated from holes that remain on the other side of the heterojunction. The short lifetime of excitons within the polymer absorber generally limits their diffusion length to at most a few tens of nanometers. Due to a rather low absorption constant, at least a few micrometers of the polymer material are, however, required to absorb a significant part of the incident radiation. The nano-structuring of the polymer | PCBM heterojunction is, therefore, needed to reconcile these two opposite requirements.^8^ Various blending techniques were developed to yield a bulk heterojunction (BHJ), where both materials are forming an inter-penetrating network, in such a way that excitons need in principle only to travel a few nanometers before they reach the interface [Fig. 1(b)]. The obtained morphology is particularly complex, as various polymer | fullerene intermixed phases can be formed. Ultrafast spectroscopy techniques are then applied to clarify how charges are generated, which involves light absorption, splitting of the exciton at a donor-acceptor junction, and separation of the ensuing electron-hole pair to charges that can be transported to the electrodes and extracted as photocurrent. At the same time, a deep understanding of the mechanisms leading to geminate and non-geminate recombination losses that compete with charge generation must be gained.

Organic photovoltaic cells based on solid-state films of solution-processed or evaporated small molecules resemble in many aspects the case of polymeric systems. A major difference, though, lies in the high extinction coefficient characterizing small dye molecules, which allows thin films of thickness <100 nm to efficiently harvest incident solar light. A much more straightforward planar heterojunction configuration can then be adopted, alleviating the need to master the complex preparation of blends with controlled morphology [Fig. 1(c)]. This configuration also simplifies the study of carrier and exciton dynamics, in particular by use of time-resolved electroabsorption (EA) spectroscopy.^5,9^

In just about four years, hybrid organic-inorganic lead halide perovskites have moved quickly from a scientific curiosity known only by a handful of specialists to the up-front of the most desirable and most studied semiconductor materials for photovoltaics. Two articles published almost simultaneously in 2012 are at the origin of what can be perceived as a global “lead rush.”^10,11^ The number of citations to these articles shows that about four thousand scientific papers have now been devoted to lead halide perovskites and the photovoltaic cells based on them. The reason for such an incredible infatuation is two-fold: This type of material is quite inexpensive and easy to produce, while the photovoltaic power conversion efficiency of simple, solution-processed solar cells has raised steeply to reach now more than 22%.^12^ In addition, the open circuit voltage for perovskite solar cells commonly approaches the thermodynamic limit by exceeding 1.2 V, making the actors of other current photovoltaic technologies quite envious. Even though the eventual commercialization of lead halide perovskite solar cells (PSCs) is not guaranteed, due to intrinsic instability and toxicity drawbacks, the field has opened up a realm of research opportunities, whose results will benefit the fundamental knowledge of relativistic semiconductors and photovoltaic systems in general, and will possibly contribute to the advent of solar cells based on new, cheap, non-toxic materials.

The discovery of perovskite photovoltaics and, to some extends, the meteoric progresses made ever since in the design of efficient photovoltaic devices can be attributed to a well-engineered serendipity. Sound experimental chemical and physical investigations, as well as theoretical calculations, however, have promptly been applied to highlight the key properties of the material. Originally conceived as mesoporous sensitized solar cells, with a conformal film of the prototype perovskite compound, methyl ammonium lead triiodide (MAPbI_3_), deposited onto nanocrystalline TiO_2_,^11^ PSCs have now evolved toward a simpler double planar heterojunction architecture. Efficient photovoltaic systems currently rely on a thin solid-state film of a mixed cation, mixed halide perovskite semiconductor, sandwiched between two donor and acceptor materials [Fig. 1(c)].^12^

Many fundamental questions regarding the key properties of lead halide perovskites have still to be addressed. Among these, the long charge carrier lifetime and diffusion length are quite intriguing. The semiconductor is indeed characterized by a strong absorption constant and a high photoluminescence (PL) quantum yield, indicative of a direct bandgap transition. The lifetime of photogenerated carriers, however, exceeds Langevin limit for direct recombination by orders of magnitude.^13^ Various possible rationales for this apparent contradiction were proposed on the basis of experimental data and theoretical predictions. Yet, no direct experimental evidence was reported that could support the idea of a phonon-assisted recombination process for photocarriers.^14^ Energy- and charge transport in multigrain films of mixed composition perovskite and electron transfer at heterojunctions are also crucial issues that still need to be addressed.

## III. DYE-SENSITIZED SOLAR CELLS

### A. Molecular engineering of sensitizers

Different strategies were employed to systematically design, synthesize, characterize, and probe new sensitizers for dye-sensitized solar cells in close collaboration between experimental and computational labs. From this joint effort, more and more panchromatic dyes with increased efficiencies are seeing the light, which shows the importance of a rational design and the synergistic effect of joint experimental and computational studies. As an evidence of the importance of molecular design in the DSSCs development, a direct relation of the electron transfer dynamics and photovoltaic performances was observed upon introducing tenuous modifications in the dyes structure. These structural alterations were not only used to tune the ability of the sensitizers to harvest the sunlight by modifying their spectra, but could also induce changes in their electronic landscape, affecting the dynamics and efficiency of charge transfer processes.

For a long time, ruthenium(II) polypyridyl complexes were the sensitizers of choice for DSSCs, due to their records in conversion efficiency.^2,4^ However, the best performing systems contained thiocyanate ligands jeopardizing chemical stability. As an alternative, a novel and promising thiocyanate-free cyclometalated ruthenium complex, bis(4,4′-dicarboxy-2,2′-bipyridine) 2-(2,4-difluorophenyl)pyridine ruthenium(II) was introduced and computationally characterized.^15^ Devices based on this sensitizer reached a remarkable power conversion efficiency of 10.1% under standard AM1.5 irradiance. Time-dependent density functional calculations showed that the absorption spectrum of this dye was characterized by an additional band in the visible region leading to a more panchromatic spectrum. Furthermore, the lowest energy transition involved a charge transfer from the ruthenium and the cyclometalated ligand to the 4-carboxylic acid-4′-carboxylate-2,2′-bipyridine moiety.

Originally, ruthenium-based sensitizers were employed in combinations with triiodide/iodide electrolyte. This 2-electron redox couple is not reversible at the surface of titanium dioxide. Direct recombination of oxidized species with conduction band electrons is hindered in this case. The use of alternative electrolytes with tunable electrochemical potential, such as cobalt-based 1-electron redox mediators, however, was prevented, due to the fast reaction of Co(III) species with injected electrons at the oxide surface. Feldt *et al.*^16^ showed that this direct recombination can be avoided in the case of organic dyes when peripheral bulky substituents were added that impede direct access of the electrolyte to the surface (umbrella effect). Such an umbrella strategy, i.e., the shielding of the dye monolayer by hydrophobic chains, was also successfully applied to Ru(II) complexes to design thiocyanate-free cyclometalated tris-heteroleptic complexes containing C_12_ alkoxy chains on the cyclometalated ligand that can be used in combinations with cobalt electrolytes.^17^ The umbrella effect in Ru(II) complexes was further investigated by *in situ* mapping of the sensitizer on a TiO_2_ surface using atomic force microscopy (AFM) measurements and molecular dynamics simulations.^18^ The latter were used to determine the energetically most favorable packing arrangements of the dyes on the TiO_2_ surface and predicted a coverage-dependent phase transition in agreement with the AFM measurements. Furthermore, the effectiveness of the umbrella effect, i.e., the shielding of the dye monolayer by hydrophobic chains, could be quantified as a function of packing density.

More recently, new types of dye sensitizers of the donor-bridge-acceptor (D-B-A) type have been introduced as promising alternatives. By considering systematic variations in the donor, bridge, and acceptor moieties, dyes can be engineered and tailored for optimal optical properties. To gain first insights into the charge-transfer (CT) properties of these systems, the excited state dynamics of *N*-phenyl piperindone-malondinitrile (DA1) was investigated as prototypical example of a donor-σ bridge-acceptor molecule.^19^ In DA1, the *N*-phenyl unit serves as the electron donor, while the dicyanoethylene acts as the electron accepting moiety and the central piperidine unit connects both via three saturated σ-bonds. This molecule is characterized by a strong charge transfer (CT) absorption band and a high fluorescence quantum yield.

The performance of time-dependent density functional theory (TDDFT) as well as the second-order approximate coupled cluster (CC2) method for this type of systems was evaluated. While CC2 and density functional theory (DFT) both predict ground state geometries that are consistent with the crystal structure, equilibrium geometries for the fluorescent charge transfer state are qualitatively different between CC2 and TDDFT. This is an example where the well-documented CT failure of TDDFT based on (semi)local functionals and kernels can affect properties of other locally excited states.^19^

Push-pull dye-sensitizers based on a donor-π bridge-acceptor (D-π-A) structure allowed to reach the best photovoltaic performances up to date. These organic dyes possess high extinction coefficients, compared with their Ru-based counterparts. Moreover, their spectral properties can be easily tuned by standard synthetic methods.^20^ Typical donors are based on coumarin, indoline, tetrahydroquinoline, triarylamine, heteroanthracene, and carbazole, while a group with the dual role of acceptor and anchoring is commonly a cyanoacrylic acid, but can be substituted with a carboxylic acid, benzoic acid, alcohol, or cyano anchor. The dependence of the charge transfer upon the donor-acceptor distance, the π-conjugation length and the coupling of the dye with TiO_2_ were studied by performing systematic structural and, therefore, electronic alterations to different series of dyes.^21^ Ultrafast pump-probe spectroscopy was used as a tool for monitoring the charge transfer within the molecule and into oxide substrates and was systematically backed by theoretical modelling using DFT calculations.^22^

A series of five D-π-A organic dye-sensitizers were investigated. The focus was set on the effect of structural modifications of the molecular architecture on the π-systems of the dyes. Two different modes of extension of the sensitizers' π-systems were investigated by means of steady-state and time-resolved spectroscopic methods. The photophysical studies of the molecules in solution and as deposited on Al_2_O_3_ or TiO_2_ films reveal that different effects on the charge-transfer characteristics evolve depending on where—within the molecular structure—the modification of the π-system is performed. Hence, the π-extension of the donor sites, for instance, leads to a strong red shift of the absorption features and a variation in light-harvesting properties. Modifying the π-bridges results in a spatial decoupling of the highest occupied molecular orbital (HOMO) from the lowest unoccupied molecular orbital (LUMO), which goes along with changes in the electronic coupling to TiO_2_. Furthermore, solution studies show that the electronic structure of the dyes governs their singlet excited-state features. The results obtained from these studies then allowed important predictions about the deactivation of the excited states of these molecules adsorbed on TiO_2_. Finally, quantum chemical methods—among others, time-dependent density functional theory calculations—provided conclusive insights into the relationship between the electronic structure of the dyes and its impact on the photoinduced charge-transfer characteristics.^23^

Modification of the anchoring group that gather the roles of physical anchor and electron acceptor was performed with UV dyes. Large effects on electron injection and back reaction were observed. The molecular modelling revealed that increasing the electron affinity of the anchor, the dipole moment is varied and increases the electronic decoupling of the HOMO and the LUMO orbitals, which in turn favors electron injection into the TiO_2_.^24^ In contrast, the regeneration process is found independent of the dye structure modification at the interface.

Two dyes based on dithieno[3,2-b:2′,3′-d]pyrrole (DTP), LP225 and LP227, were synthesized, characterized, and compared with CPDT, a well-known analogue having a high synthetic cost and less versatility.^25^ In both cases, the characteristics of the first excited state in terms of single particles orbitals can be characterized as HOMO-LUMO transition corresponding to an intramolecular charge transfer. The electronic push through donor-π-acceptor systems can be enhanced with a fluorine donor bridged by a cyclopentadithiophene to a cyanoacrylic acid acceptor in the dye JF4199.^26^ This sensitizer showed performances superior to previous organic dyes with a conversion efficiency of 10.3% in the presence of a cobalt electrolyte.

**FIG. 2.**
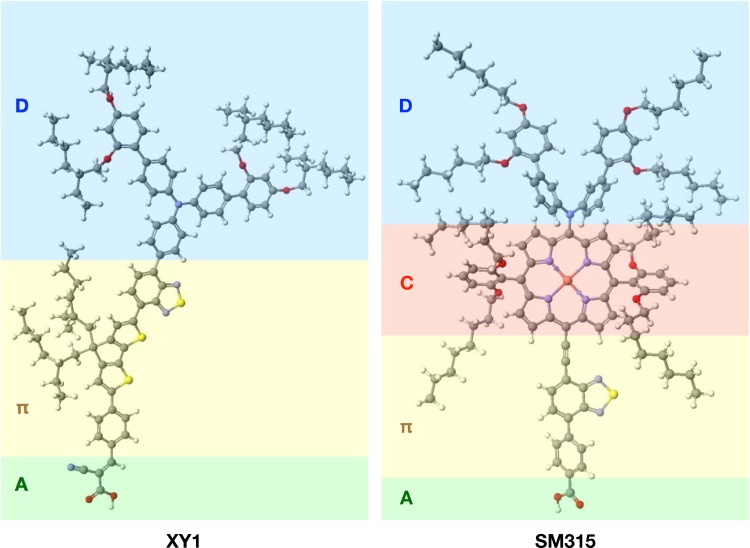
Molecular structures of exemplary XY1 and SM315 push-pull organic dye-sensitizers discussed in the text. Color-code for atoms: C (dark grey), H (white), O (red), N (blue), S (yellow), Zn (orange). In the case of the SM315 molecule, the zinc porphyrin chromophore (C) is intercalated between the triarylamine donor moiety (D) and the π-conjugated bridge (π).

Liquid electrolytes and solid-state hole-transport media (HTM) based on the Cu^II^/Cu^I^ redox couple were recently introduced that offer a better potential match with the HOMO of optimized sensitizers.^27^ Combining two different D-π-A dyes with complementary absorption spectra, coded D35 and XY1 (Fig. 2), and a liquid electrolyte containing the copper complex Cu(II/I)(tmby) as a redox shuttle, a DSSC achieved a power conversion efficiency of 28.9% under illumination from a model warm-white fluorescent light tube.^28^ A record 11% power conversion efficiency was obtained by using a hole-transport material composed of an amorphous solid blend of [Cu^II^(tmby)_2_](TFSI)_2_ and [Cu^I^(tmby)_2_](TFSI) infiltrated in a mesosopic TiO_2_ layer, sensitized by the Y123 organic dye-sensitizer.^29^

Currently, the highest performing sensitizers belong to the class of (biomimetic) porphyrin-based D-π-A molecules. Porphyrins are characterized by strong light absorption due to the Soret and Q-bands, but with little to no light capture between these two regions. Important efforts have therefore been made to design dyes with panchromatic light harvesting abilities via systematic variations and computational design of the donor, acceptor, or bridge components. In an attempt to vary the donor moiety, an ullazine unit was employed with the porphyrin as π-bridge to design the novel dye SM63.^30^ The UV-vis absorption spectrum showed an important perturbation of the Sorbet and Q-bands due to the ullazine's substantial electron donating character leading to an improved absorption of green and red light compared with other D-π-A porphyrin-based dyes. However, the dye performed poorly when used in a DSSC with iodide electrolyte yielding a conversion efficiency of only 7.35%. This could be explained by undesired aggregation of the dye in solution due to its pronounced hydrophobic nature.

Modifying the electron accepting moiety, a panchromatic sensitizer compatible with a cobalt redox couple was obtained with the SM315 porphyrin dye where a proquinoidal benzothia-diazole (BTD) unit was incorporated (Fig. 2). Combining the findings from systematic variations of donor, bridge and acceptor, it was possible to design a new sensitizer with the currently top photovoltaic conversion efficiency of 13.0% under solar AM1.5 irradiance.^31^ These findings were predicted and rationalized by linear response TDDFT calculations that showed that the HOMO was localized on the donor and was not disturbed by the choice of the acceptor. The LUMO showed an important localization toward the BTD moiety of the acceptor explaining the enhanced CT character of the HOMO-LUMO transition.

### B. Charge injection and carrier dynamics in wide bandgap metal oxides

Electron injection from a photoexcited molecular sensitizer into a wide-bandgap semiconductor is the primary step toward charge separation in dye-sensitized solar cells. According to the current understanding of DSSCs functioning mechanism, charges are separated directly during this primary electron transfer process, yielding hot conduction band electrons in the semiconductor and positive holes localized on oxidized dye molecules at the surface. Ultrafast X-ray absorption and THz spectroscopies measurements carried out in the frame of the NCCR-MUST project, however, upset this simple view.

Time-resolved fluorescence techniques at high time resolution were applied to the Ru(II) polypyridil complex sensitizer in solution, adsorbed on the surface of redox-inactive Al_2_O_3_ and on TiO_2_. Injection of an electron from the photoexcited complex into the latter semiconductor was found to occur in <10 fs.^32^ Picosecond X-ray absorption spectroscopy was then demonstrated to probe with element-selectivity (Ru and Ti atoms) the fate of electrons in dye-sensitized and bare anatase titanium dioxide. Evidence of injection or electron delivery was given by its trapping at defects, which were on the outer surface in the injection case, or buried deep in the surface shell in the case of band gap excitation of the oxide.^33^ The trapping time of the electron in the bare TiO_2_ was probed for the first time with femtosecond resolution and showed to occur in <200 fs, reaching the conclusion that the injected electron is trapped at or very near the unit cell where it was created.^34^ The detection and identification of hole-trapping in ZnO were recently achieved using ps and fs X-ray absorption and emission spectroscopies.^35,36^

X-ray tools monitor mainly charge trapping processes, while optical methods (IR, visible-UV) tend to restrict to the detection of the featureless free carrier absorption in the conduction band (CB) of the material. Hence, it is important to have an additional table-top experimental method available that could ensure substrate-specific diagnostic. Following a very detailed characterization of the electronic absorption spectrum of anatase TiO_2_ using deep-UV spectroscopy,^37^ it was shown that the first excitonic transition undergoes Coulomb screening upon electron injection from a sensitizer, which gives rise to distinct features in the deep-UV transient absorption (TA) spectrum.^38^ This approach turns out to be ideal for any combination of substrate/sensitizers, including solid ones, as the free carrier absorption is minimal in the deep-UV. It has allowed recently to discern the electron injection process in the case of Au nanoparticles on TiO_2_ and N719 on ZnO.

**FIG. 3.**
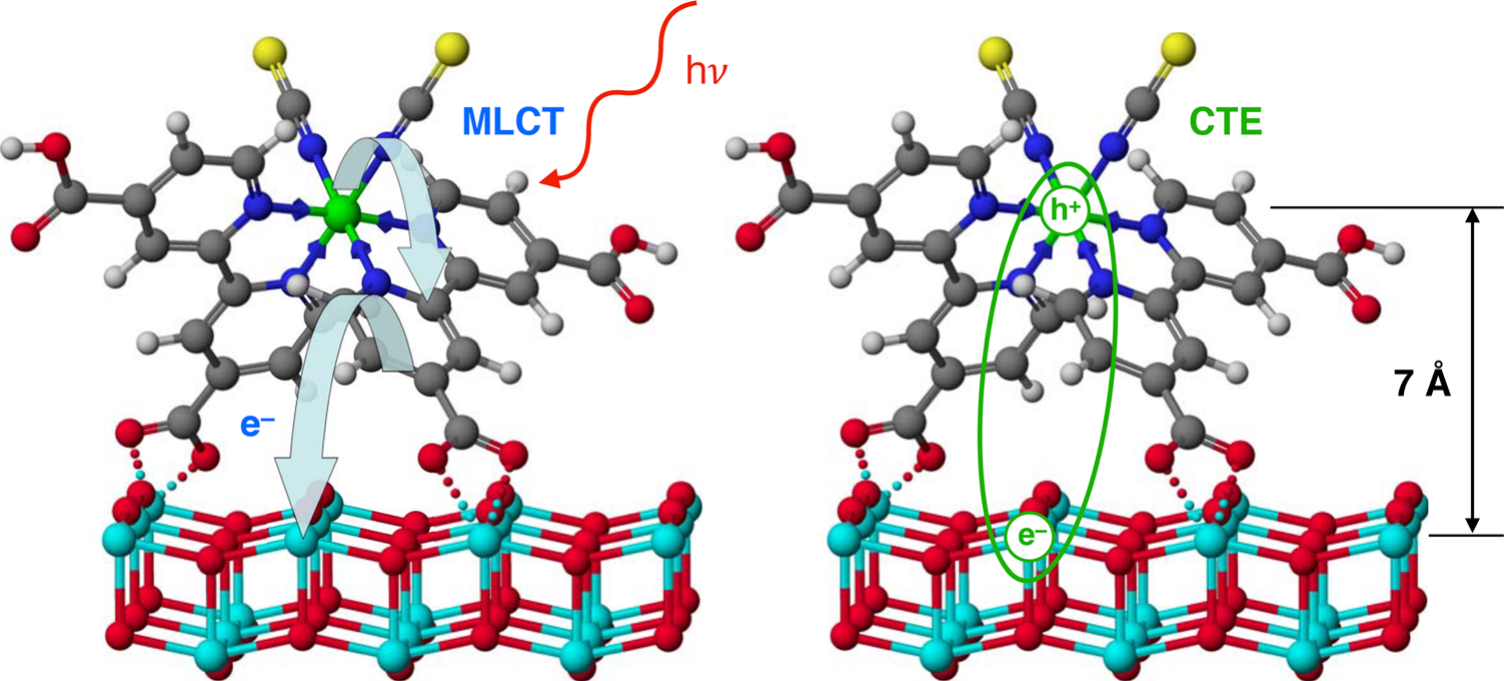
Ball-and-stick molecular model of N719 dye-sensitizer adsorbed on the TiO_2_ anatase (101) surface. Ultrafast electron injection into the solid oxide takes place upon photoexcitation of the Ru(II) complex by a metal-to-ligand charge transfer (MLCT) transition. Provided the excess excitation energy is not too large (Δ*E*_ex_ < 1 eV, λ_pump_ ≥ 470 nm), a charge transfer exciton (CTE) is formed through Coulombic interaction between the positive charge (h^+^) located within the HOMO of the oxidized dye cation and the injected electron (e^−^) trapped at a neighboring, uncoordinated surface Ti(IV) site, with an e^−^-h^+^ separation distance ≥7 Å.

Transient optical absorption measurements applied to mesoporous semiconducting films of TiO_2_ coated by a monolayer of a Ru^II^ heteroleptic complex dye, allowed to monitor the appearance of the dye cation at the interface directly with a typical time constant <50 fs, in accordance with ultrafast fluorescence up-conversion measurements. Significantly slower charge injection into nanocrystalline titanium dioxide was witnessed for the plant-pigment betanine^39,40^ and the D-π-A organic dye-sensitizer Y123,^41^ where the interfacial charge transfer process was observed to take place with time constants of 6–8 ps and 2 ps, respectively. The conclusion provided by X-ray absorption experiments that electrons can be injected directly into trap states in the proximity of the dye-sensitizer molecules they originate from, rather than escaping quickly as hot carriers has important consequences. Coulomb attraction between the trapped electron and the positive charge left on the dye molecule can give rise to an interfacial charge-transfer exciton (CTE) (Fig. 3). CTE cannot be detected optically. However, the formation of such a bound electron-hole pair was evidenced using time-resolved THz spectroscopy (optical pump-THz probe, OPTP) applied to the same samples.^42^ CTE formed across the interface indeed prevent a majority of injected charges to contribute to the transient THz photoconduction. The mobility of photo-injected conduction-band electrons in TiO_2_ nanoparticles was found to increase markedly when anions were adsorbed onto the surface, screening the Coulomb interaction and decreasing the exciton binding energy upon local reorganization with a typical time-constant of 200–300 ps. CTE formation and dissociation depend on the excess kinetic energy of the transferred electrons or on the vibrational energy of the cations and, therefore, on the excess excitation energy of the sensitizer. As a result, the yield of CTE formation was observed to decrease for shorter excitation pump wavelengths and even to become negligible for λ_ex_ < 460 nm.^43^

This finding highlights the importance of dielectric screening by ions in carrier separation, which is of particular importance in solid-state dye-sensitized solar cells, where the hole transport medium does not intrinsically contain charges. They shed new insights into the possible mechanism of action of dopants, such as in particular, Li^+^ and H^+^ cations. They show that the existence of charge transfer states is a common property of liquid electrolyte-based and solid-state dye-sensitized solar cells. Finally, they suggest a unified picture for free carrier generation and separation in OPV and hybrid organic-inorganic photovoltaic systems using wide band-gap semiconductors, where the involvement of interfacial charge transfer excitons and charge transfer states represents a paradigm shift.

## IV. ORGANIC PHOTOVOLTAIC SYSTEMS

### A. Charge separation in small molecule-based OPV systems

In spite of numerous investigations, electron mobility values and dynamics in PCBM and PCBM-based OPV devices have remained unclear, preventing strong understanding and modeling of the device performance. Time-resolved electroabsorption spectroscopy was employed in combination with photocurrent measurements for the investigation of the charge drift dynamics in thin films of pristine PCBM and in its blend with a merocyanine molecular semiconductor (MD376: PCBM blend).^44^

The experimental scheme is based on a femtosecond pump-probe laser setup utilizing white light continuum probe pulses. The application of a pulsed voltage (0.1–20 V, square pulses, 100 *μ*s width, 500 Hz repetition rate) onto a thin layer sample (typically <100 nm thick) between a transparent conductive metal oxide electrode and an evaporated metal contact imposes a modulated electric field, which intensity is typically >10^8^ V m^−1^. Electro-modulated differential absorption (EDA) spectra recorded in reflectance mode result from induced Stark shifts, which intensity and direction vary for different absorption bands. The screening of the external electric field sensed by the material by drifting photogenerated charge carriers results in the gradual attenuation of the Stark effect. The technique allows for monitoring the separation distance between charge carriers of opposite sign with sub-100 fs time-resolution.

The role of charge screening was examined and this effect was shown to lead to a qualitatively different interpretation of the electron mobility dynamics. It was then concluded that electron mobility is time-independent and weakly dispersive, however, strongly field-dependent in pristine PCBM films. On the contrary, the charge mobility is substantially lower and experiences rapid relaxation in blends with an organic donor material.^44^

The charge pair separation dynamics in the latter experiment was convoluted with the complex three-dimensional motion of already separated charge carriers in the disordered bulk heterojunction and, hence, limited conclusions could be drawn from these measurements. Formation of interfacial charge transfer excitons, and their dissociation into free charge carriers in model planar heterojunction systems were more rarely addressed. Because of precisely known geometrical parameters and a well-defined geometrical plane where separation of the charges takes place, CTE dynamics and free carriers separation in bilayers are expected to be less entangled. The averaged microscopic displacement of charges at the planar heterojunction results in the macroscopic perturbation of the electric field in the donor and in the acceptor layers. In addition, transports of photogenerated electrons and holes are spatially separated. Thus, planar heterojunction solar cells may serve as an ideal model system for the investigation of the charge generation phenomenon.

**FIG. 4.**
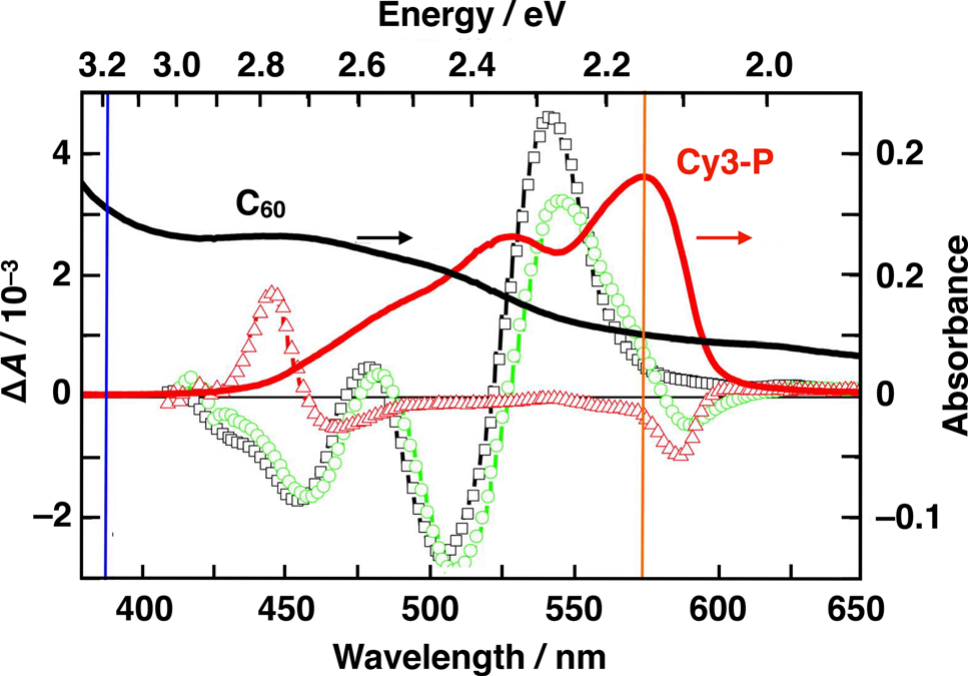
Electroabsorption spectra of pristine C_60_ (black squares, 50 nm-thick film), Cy3-P cyanine dye (red triangles, 25 nm-thick film), and C_60_ | Cy3-P planar bilayer (green circles, 30 nm | 20 nm-thick layers). The applied voltage was 2 V for all films. Absorption spectra of pristine 20 nm-thick films are also shown (right scale, solid lines). Vertical lines show both 390 and 575 nm excitation wavelengths chosen for selective excitation of C_60_ and Cy3-P, respectively.

The carrier escape from the Coulomb attraction and the dynamics leading to free charge carriers have not been addressed explicitly until recently, probably because of the absence of appropriate experimental methods capable of disclosing the carrier motion within organic photovoltaic devices with a sufficient time resolution. The free carrier formation process in a planar bilayer OPV cell based on the heterojunction between a solid thin film of the cyanine dye CY3-P and evaporated C_60_ as an acceptor material was studied by using ultrafast electroabsorption spectroscopy.^45^ The important advantage of the transient Stark effect technique when applied to planar heterojunction solar cells is that it allows to distinguish between the electron and hole motions by analyzing different spectral regions, as long as electroabsorption (EA) of the donor and acceptor materials are spectrally separated (Fig. 4). Moreover, fast CT state formation, fast electron motion in thin fullerene layer, and close to one-directional carrier motion allowed to distinguish between CTE dynamics, Coulomb bound charge pair separation, and carrier drift processes. It was shown that only charge pairs with an effective electron-hole separation distance of less than 4 nm are created during the dissociation of Frenkel excitons upon ultrafast charge transfer at the interface. The dissociation of the Coulomb bound charge pairs was identified as being the rate-limiting step for charge carriers' generation. Interfacial CTE split into free charges on the timescale of tens to hundreds of picoseconds, mainly by electron escape from the Coulomb potential over a barrier that is lowered by the electric field. The motion of holes in the small molecule donor material during the charge separation was found to be insignificant.^46^

### B. Carrier and exciton dynamics in polymer bulk heterojunctions

Exciton dissociation by charge separation in polymer: fullerene blends has been reported to occur on the ultrafast time scale, in less than 100 fs.^47^ This has raised considerable questions, since such a fast time scale is not consistent with exciton diffusion through neat polymer domains over a distance of about 10 nm, in order to reach a PCBM quenching site. One hypothesis that has been suggested is that excitons are highly delocalized for about 100–200 fs after photoexcitation, so that they sample a larger extent of the BHJ, and that their dissociation takes place before the excited state relaxation to a more localized species.^48–50^ Subsequently, it was shown, using femtosecond transient absorption (TA) studies on polymers such as poly[2,5-bis(3-tetradecylthiophen-2-yl)thieno[3,2-b]thiophene] (PBTTT), poly[[5-(2-ethylhexyl)-5,6-dihydro-4,6-dioxo-4H-thieno[3,4-c]pyrrole-1,3-diyl][4,8-bis[(2-ethylhexyl)oxy]benzo[1,2-b:4,5-b′]dithiophene-2,6-diyl]] (PBDTTPD) and poly(3-hexylthiophene-2,5-diyl) (P3HT), that an even more important parameter leading to ultrafast exciton dissociation in polymer:fullerene blends is the presence of an intermixed polymer:fullerne phase.^51–55^ Prompt (∼100 fs) exciton dissociation was found to occur predominantly in the regions where the polymer and fullerene are molecularly intermixed, so that no exciton diffusion is necessary. On the other hand, a slower (delayed) exciton dissociation is observed, if the excitons need to diffuse through neat domains to a quenching site.

**FIG. 5.**
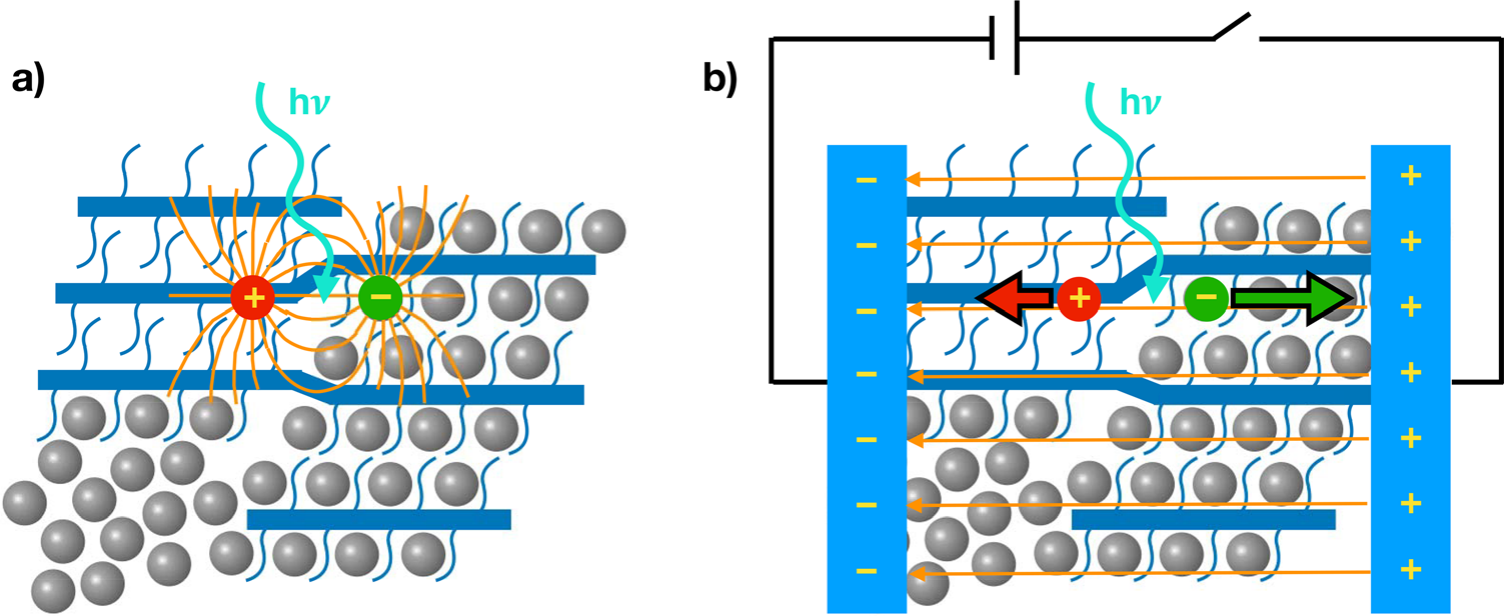
Cartoon representing a bulk heterojunction composed of neat PCBM phase (grey spheres), neat pBTTT phase (blue rods), and intermixed polymer:fullerene phase. (a) Photo-generated charges create a local electric field around them, which induces an electro-absorption signal in the transient absorption spectra, allowing to determine in which phase the charges are found. (b) A bulk electroabsorption response is induced by an external electric field, which is shielded by the transport of photo-generated charges to the electrodes, allowing to deduce the spatio-temporal separation of the electron-hole pair.

Once the excitons have dissociated at a polymer: fullerene domain-interface or molecular boundary in the intermixed regions, the generated electron-hole pairs can either spatially separate to free charges, or undergo geminate charge recombination to the ground state. The detailed mechanism of free charge generation in organic solar cells is still highly debated, with conflicting accounts of ultrafast free charge generation,^56–61^ and of slowly separating CT-states.^62–64^ The ultrafast free charge generation has been justified by long-range charge separation,^56,58,59^ by delocalization into neat domains,^57^ and by the contribution of hot states.^50^ On the other hand, the dissociation of relaxed CTE has been related to high local charge carrier mobility.^62^ Using pBTTT: PCBM blends with different arrangements of neat and intermixed phases (different phase morphologies), it was shown by transient absorption spectroscopy (TAS) that geminate charge recombination occurs predominantly in the intermixed regions, but that it can be prevented by the presence of nearby neat pBTTT or PCBM domains.^51–53^ Moreover, a pronounced electro-absorption (EA) signal in pBTTT was exploited, allowing to directly visualize the transport of electrons and holes between the different phases of the blend. On the one hand, the EA due to local electric fields of photogenerated charges was extracted from the TA spectra,^51–53^ and on the other hand, the EA was induced by an external bias in the electro-modulated differential absorption (EDA) spectroscopy, which has allowed to time-resolve the spatial electron-hole separation (Fig. 5).^51,65^ Reconciling opposing views found in literature, it was demonstrated that the fate of photo-generated electron–hole pairs—whether they dissociate to free charges or geminately recombine—is determined at ultrafast times, despite the fact that their actual spatial separation can be much slower. Whether the electron-hole pairs are able to dissociate or not appeared to be instantaneously determined at the moment of their generation (by the molecular arrangement and local environment of the charge pairs, which mainly affect their electronic coupling). However, the spatial dissociation of the charges to reach the ∼5 nm electron-hole separation needed to overcome their Coulomb attraction is relatively slow, and can take 5–30 ps, depending on the phase morphology.

## V. PEROVSKITE PHOTOVOLTAICS

### A. Structural, electronic, and transport properties of the material

Mixed organic/inorganic or fully inorganic lead trihalide perovskites attract currently broad attention since they have recently emerged as highly efficient light harvesting and charge carrier transport materials for solar cells,^11,66–70^ with rapidly evolving record power conversion efficiencies.^71^ In fact, they exhibit most of the ideal features required for photovoltaic applications, demonstrating properties as either *n*-type or *p*-type semiconductors with close to optimal bandgaps for solar energy conversion. These favorable characteristics coupled with the ease of fabrication by solution processing leading to low production costs make them excellent candidates as optoelectronic materials.^72–81^

Broad optical absorption is the key to the outstanding performance of perovskite-based solar cells. MAPbI_3_, for instance, has a direct experimental band gap of 1.55 eV, which makes this material a good light absorber over the whole visible solar emission spectrum. However, the objective and still an open issue in the field of organic-inorganic halide perovskites is to obtain a compound with a band gap close to the ideal value of 1.3–1.4 eV.^82^ Apparently, changes in the chemical composition and variations in the structure of the perovskite can tune the bandgap over a wide range. This flexibility is corroborated by experimental results and can be rationalized theoretically by establishing a relation between the chemical composition, crystal structure, and the electronic properties of the perovskites via two key parameters: the overlap between the metal and halide orbitals and the charge of the divalent cation.^83^ Through density functional theory band structure calculations, it has been observed that the atomic orbital composition of the valence band and conduction band close to their edges remains largely unchanged over a wide range of different chemical and crystallographic variations. Indeed, the valence band of perovskites of the general formula ABX_3_ is formed by an antibonding combination of B (divalent cation) ns and X (anion) mp orbitals, having a rather covalent character. The conduction band is also characterized by an antibonding combination, this time by B (divalent cation) np and X (anion) ms orbitals, having a more ionic character. Chemical and structural changes that increase the negative overlap between orbitals of B and X ions thus result in a shrinking of the band gap.

An effective mass picture provides quantitative predictions for the efficiency of the charge carrier transport. Ashari-Astani *et al.*^84^ studied the charge carrier properties of halide perovskites, suggesting a strong correlation of the bandgap with the effective masses showing that the lower the band gap is, the lower the effective masses are. However, calculations in a variety of compounds with different chemical compositions and crystal structures and by introducing defects and dopants showed that the effective masses are not changing significantly, maintaining the good transport properties of perovskite materials.

The origin of the observed hysteresis of the current-voltage (IV) curve of MAPbI_3_-based solar cells is a long-standing issue in this field, since this effect complicates the determination of the “real” solar to electrical power conversion efficiency making “bad cells look good.”^85–88^ Two hypotheses have been put forward trying to explain the origin of this phenomenon. According to this, hysteresis is observed either due to possible ferroelectric effects that are caused by the orientation of the organic cations,^89,90^ or due to the movement of ionic species.^86^ Meloni *et al.*^91^ suggested that hysteresis is due to halide ion vacancy migration induced polarization of the perovskite layer, and were able to exclude a possible ferroelectric effect due to the alignment of the methylammonium (MA) ions as the source of hysteresis. Simulations that are performed to examine the ferroelectric effect showed that the polarization due to dipole alignment takes place on the picosecond time scale, which is too short to account for the observed IV hysteresis that can be associated with a process with a characteristic time in the milliseconds-to-seconds range. On the other hand, the longer time scale of vacancy migration makes this phenomenon a more possible candidate as the source of the observed IV hysteresis.

**FIG. 6.**
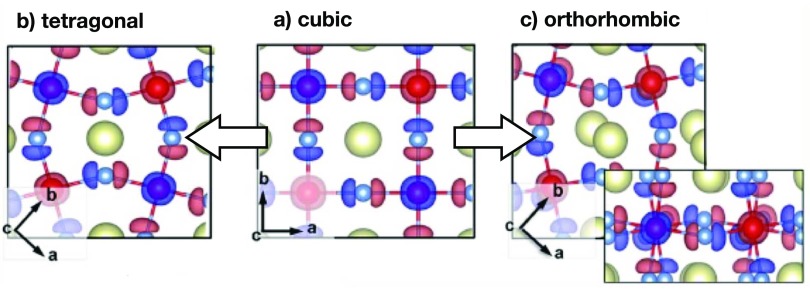
Valence band maximum (VBM) (blue frame) and conduction band minimum (CBM) (red frame) orbitals of CsPbI_3_. Panels (a)–(c) show how the tilting of PbX_6_ octahedra affects the overlap.

To gain a better understanding of the photophysical processes occurring in perovskite materials, it is important to also explore them at low temperatures where the additional complexity induced by thermal effects is minimized. Dar *et al.*^92^ studied the origin of the peculiar band gap shift as a function of temperature and the dual emission in organic-inorganic halide perovskites. From DFT calculations that have been performed for MAPbI_3_, the additional photoluminescence peak can be assigned to the presence of molecularly disordered orthorhombic domains. This supports the hypothesis that the two photoluminescence peaks are associated with methylammonium-ordered and methylammonium-disordered domains in MAPbI_3_. It is worth emphasizing that the disordered domains are not tetragonal inclusions, but rather orthorhombic domains with a molecular disorder. Between 120 K and 150 K, i.e., prior to the orthorhombic to tetragonal phase transition of MAPbI_3_, two phenomena can be observed experimentally. The higher energy photoluminescence peak disappears and the lower energy peak smoothly shifts toward even lower energies. The disappearance of the high-energy emission peak can be associated with the rotational mobility of the MA cations in the tetragonal phase of MAPbI_3_. Concerning the evolution of the low energy emission peak, the mobility of methylammonium cations in disordered orthorhombic domains gradually increases with temperature, and eventually leads to a smooth transition into a regular tetragonal phase (Fig. 6).

Notwithstanding the fact that organic/inorganic trihalide perovskites exhibit most of the desired properties for solar cells applications, there are still open issues that have to be addressed. The long-term stability is one of them, and is complicated by the fact that various crystalline phases exist in a narrow temperature range. The main phases that are observed are a trigonal structure called α phase or black phase and a hexagonal structure called δ phase or yellow phase.^93^ The latter, however, is not suitable for solar cell applications. Therefore, one of the main questions is how one can differentially stabilize the 3D perovskite-phases. To this end, it has been reported that mixing different monovalent cations such as methylammonium (MA), formamidinium (FA), cesium (Cs^+^) and different anions such as iodide and bromide, leads to an increase in the stability of the perovskite phase.^12,94–98^ Nowadays, since the pure perovskite compounds fall short in some aspects mainly due to thermal or structural instabilities, it has become an important design principle to mix cations and halides in order to achieve compounds with improved structural and thermal stability.

**FIG. 7.**
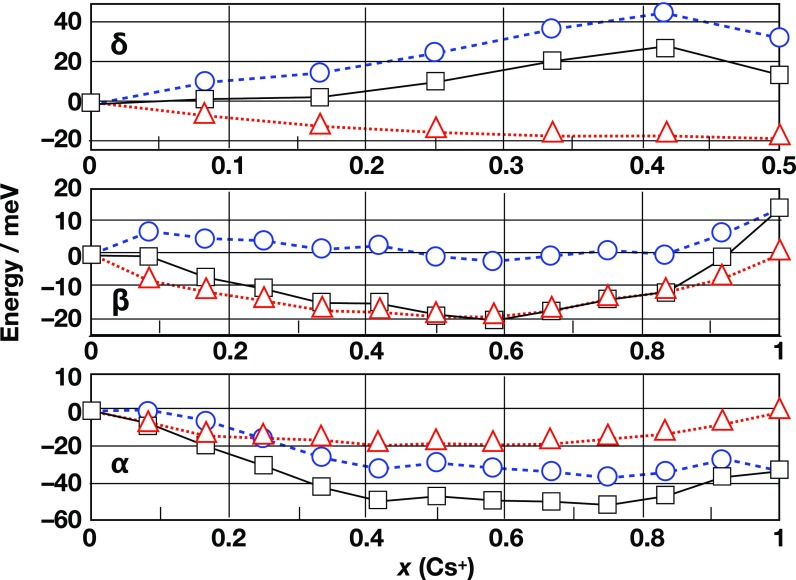
Variation of internal energy Δ*E* (blue circles and dashed line), mixing entropy contribution, −*T·*Δ*S* (red triangles and dotted line) and Helmoltz free energy Δ*F*=Δ*E* – *T*·Δ*S* (black squares and solid line) as a function of Cs^+^ content in the mixed cation Cs_x_FA_(1–x)_PbI_3_ perovskite.

Yi *et al.*^96^ proposed an MA-free mixed cation perovskite, Cs_x_FA_(1-x)_PbX_3_ as an attractive material for perovskite solar cells applications exhibiting both high efficiencies and good thermal (phase) stability. This could be rationalized by the fact that the atomistic structures of the two δ phases of CsPbI_3_ and FAPbI_3_ differ significantly, since the δ phase of CsPbI_3_ consists of edge-sharing octahedra, and the δ phase of FAPbI_3_ consists of face-sharing octahedra. On the other hand, the atomistic structures of both α and β perovskite phases consist of corner-sharing octahedra. Even from this simple argument, one can suggest that in the case of α and β perovskite phases, the mixing is favorable, while it is unfavorable in the case of the non-perovskite phases. This statement has been proven using thermodynamic arguments. The free energy of the pure compounds as well as the one of the mixed compounds has been estimated as the sum of the enthalpic contribution and the mixing entropy contribution (Fig. 7). The energetic analysis of the δ phase shows that replacement of the organic cation FA by the inorganic cation Cs^+^ leads to a significant destabilization with respect to the pure FAPbI_3_ and CsPbI_3_ δ phases. The mixing entropy contribution cannot compensate for this penalty; hence, cation mixing cannot take place in the δ phase. On the contrary, in *α* and *β* perovskite phases, the negative value of the free energy change upon mixing denotes an improved stability of the mixed cation perovskite. Therefore, within this model, the transition temperature from δ to α and β perovskite phases is reduced by 200 K–300 K when going from the pure FAPbI_3_ to the mixed systems, which explains why the perovskite phase is stable at room temperature for the mixed compound. Thus, there is a preferential stabilization of the perovskite phase over the non-perovskite δ phase by mixing two compounds that have structurally different δ phases. More recently, Syzgantseva *et al.*^98^ were able to show that the incorporation of Rb^+^ and Cs^+^ cations is able to stabilize the perovskite phase at lower temperatures than methylammonium, thus facilitating the formation of an unannealed precursor close to a homogeneous perovskite phase.

### B. Carrier dynamics in nanoparticles and thin films

**FIG. 8.**
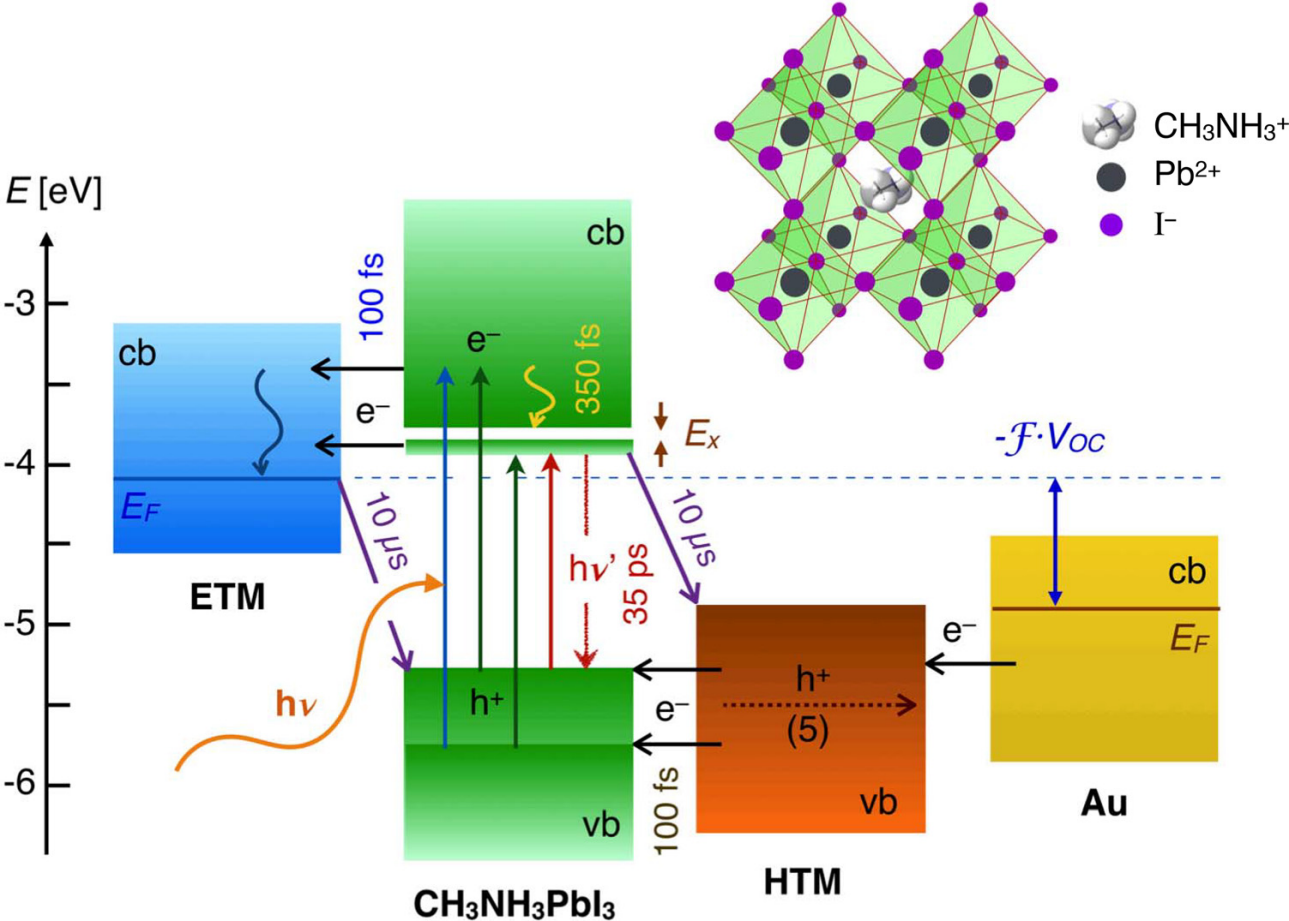
Schematic diagram of energy levels and electron transfer processes in a TiO_2_ | perovskite | HTM cell. The structure of the cell unit of the MAPbI3 perovskite in its cubic form is shown in the top right illustration.

Ultrafast transient absorption spectroscopy and time-resolved terahertz photoconductivity measurements provided important insights into the charge transfer processes taking place in TiO_2_ and Al_2_O_3_ mesoporous films impregnated with MAPbI_3_ perovskite and the organic hole-transporting material (HTM) *spiro*-MeOTAD (Fig. 8).

**FIG. 9.**
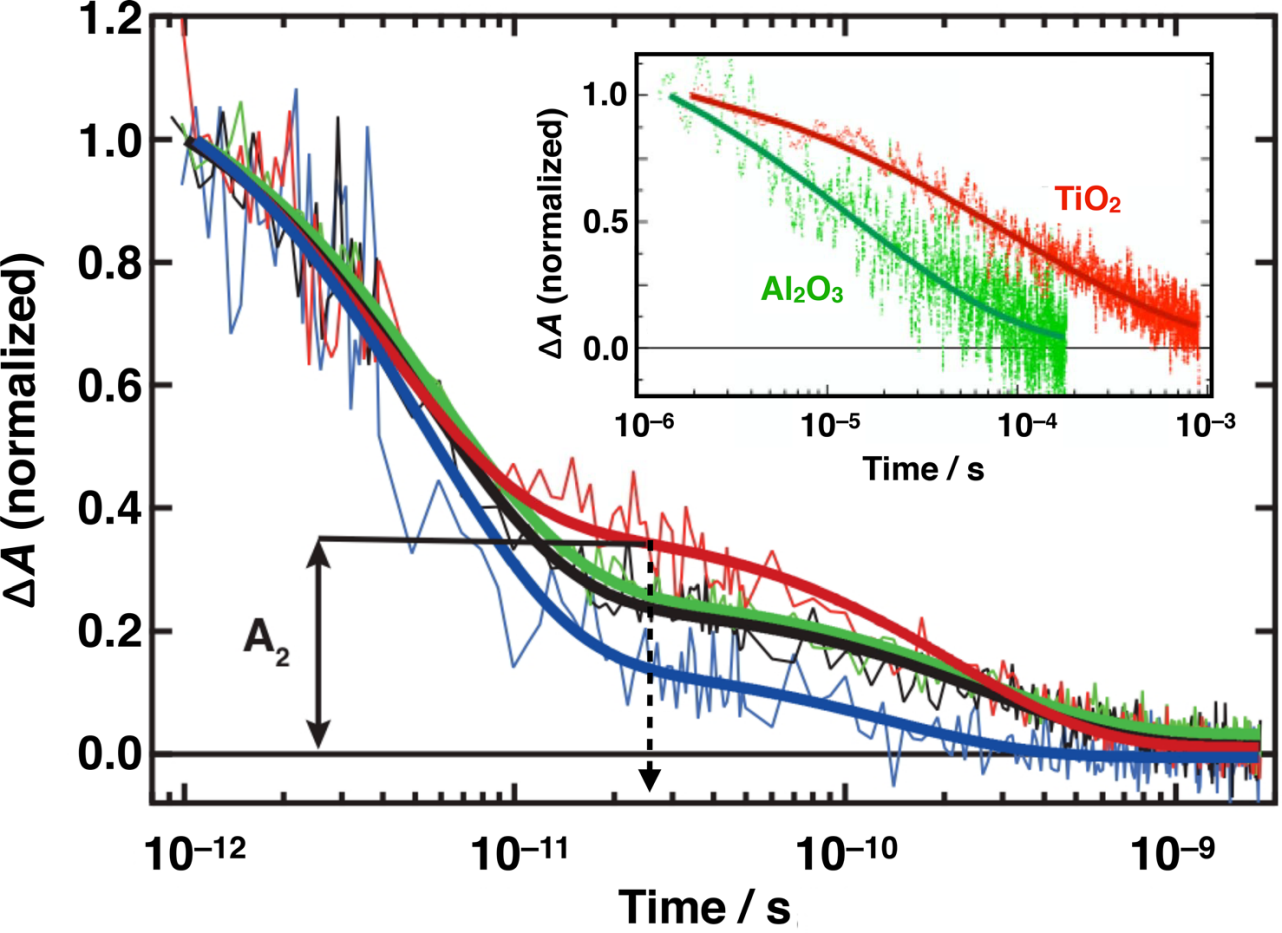
Time evolution of electron and hole populations in photoexcited MAPbI_3_ perovskite in various systems: CH_3_NH_3_PbI_3_ | TiO_2_ (black); CH_3_NH_3_PbI_3_ | Al_2_O_3_ (blue); *spiro*-MeOTAD | MAPbI_3_ | TiO_2_ (red); *spiro*-MeOTAD | CH_3_NH_3_PbI | Al_2_O_3_ (green). Thick solid lines represent bi-exponential fits of experimental points starting at *t* = 1 ps. A_2_ represents the normalized absorbance change at 25 ps, used as a metric to compare the various samples. Inset: Charge recombination dynamics obtained from nanosecond laser flash photolysis of the same samples. Signals mainly reflect the decay of the h^+^(HTM) population. Solid lines represent stretched exponential fit of experimental data. All transient absorption signals were monitored at a probe wavelength *λ* = 1.4 *μ*m following pulsed excitation at 580 nm.

Marchioro *et al.* monitored the transient absorption in the near infrared of photogenerated charge carriers in the perovskite.^92^ Results showed that the decay of the carrier population due to recombination is markedly slowed down upon infiltration of the hole-transporting material, which is consistent with primary hole-injection from the photoexcited perovskite into the HTM. Evidence for ultrafast electron injection from MAPbI_3_ into the TiO_2_ film was found as well. In photovoltaic systems based on a TiO_2_ | perovskite | HTM architecture, it was shown then that primary charge separation occurs at both junctions with the electron-transporting oxide and the HTM simultaneously, with ultrafast electron- and hole-injection taking place from the photoexcited light-absorbing semiconductor within similar timescales (Fig. 9).^99,100^

Time-resolved terahertz spectroscopy (TRTS) is a method of choice to study the carrier dynamics in semiconductors and at heterojunctions that are central to optoelectronic systems. The technique allows in particular to probe the dynamics of the complex conductivity of a material. Combined with transient absorption spectroscopy, able to provide a direct measurement of the carrier density, TRTS affords a handle for monitoring the time-evolution of charge mobility and to distinguish between free carriers, excitons, interfacial charge transfer states, polarons, and trapped electrons and holes.

Time-resolved microwave and THz experiments support the results obtained by transient near infrared absorption spectroscopy. The photoconductivity of the active layer arises almost exclusively from carriers in the perovskite material. The effect of the excitation wavelength upon the transient THz absorption amplitude and spectrum allowed evidencing the formation of excitons, polarons, and bipolarons during the first picoseconds following pulsed excitation of the perovskite, on a time-scale slower than interfacial charge transfer. We thus conclude that the exceptionally large carrier diffusion lengths in the semiconductor (100 nm–1 *μ*m) allow photogenerated electrons and holes to reach the selective contacts separately (TiO_2_ and the HTM, respectively), where they are readily injected, before they could associate in the form of excitons and eventually recombine. This unique charge separation mechanism unveiled by using ultrafast laser spectroscopy makes perovskite hybrid solar cells a new type of photovoltaic converter of its own and a new realm of scientific investigation and technological development.^100–102^

Ponseca *et al.* used a combination of spectroscopic techniques, namely, photoluminescence (PL), transient absorption (TA), time-resolved terahertz spectroscopy (TRTS), and time-resolved microwave conductivity (TRMC), to monitor light-induced processes in MAPbI_3_ perovskite from the subpicosecond to a hundred of microsecond time scale.^103^ This work focuses on the TRTS study of fast carrier dynamics in neat MAPbI_3_ and when a layer of mesoporous TiO_2_ and Al_2_O_3_ is filled with perovskite. The remarkable signal to noise is mainly obtained, thanks to the prior development of high energy single cycle THz source via optical rectification, which led to as much as 30 *μ*J per pulse at 100 Hz^104^ and 175 *μ*J at 10 Hz.^105,106^

**FIG. 10.**
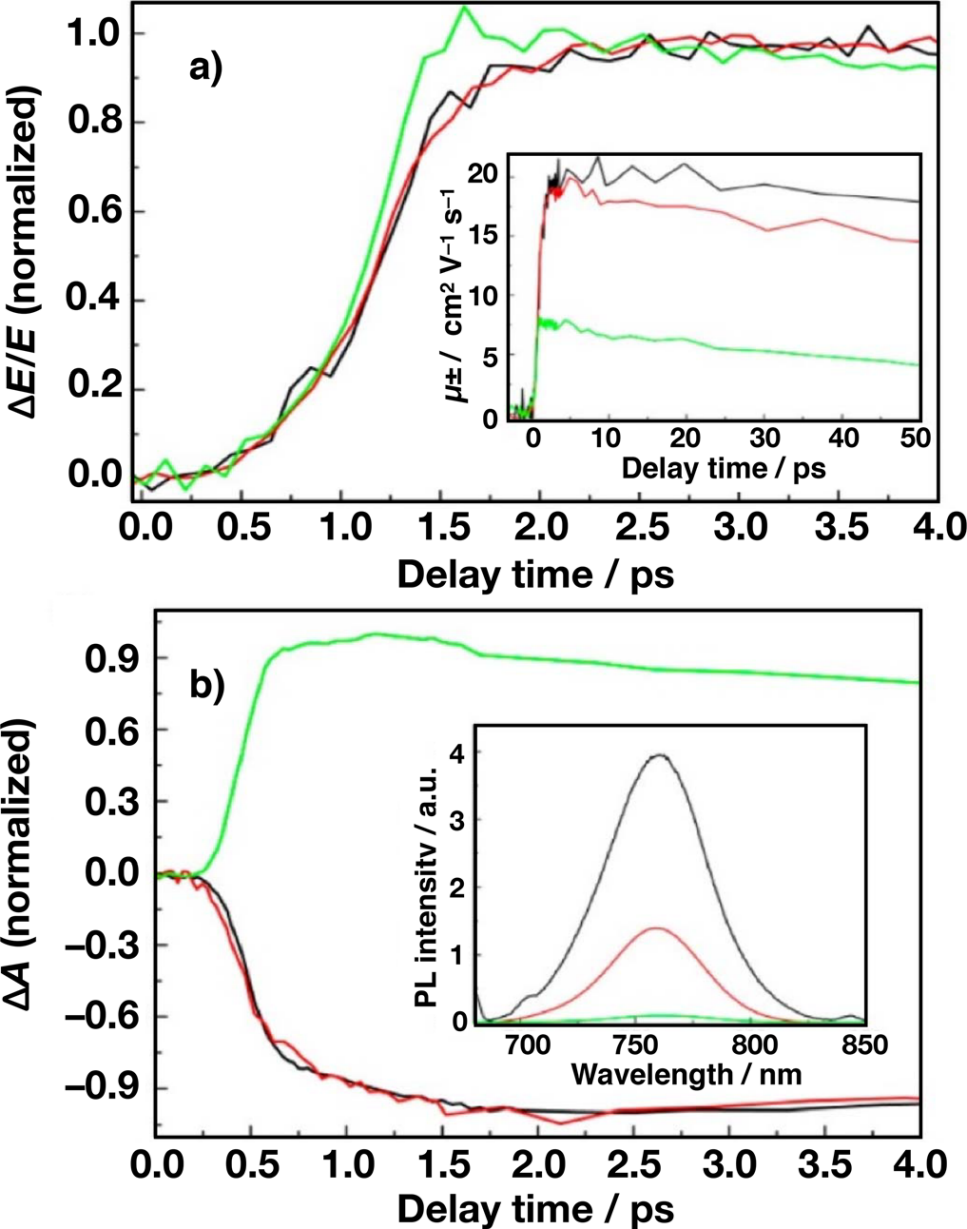
Early time dynamics of neat MAPbI_3_ (black), MAPbI_3_ | Al_2_O_3_ (red), and CH_3_NH_3_PbI_3_ | TiO_2_ (green). (a) THz kinetics (*λ*_pump_ = 400 nm, *I*_exc_ = 1.7 × 10^13^ photons/cm^2^ per pulse) normalized to 1. Inset shows the time-evolution of the electron mobility for the first 50 ps, obtained from the measured THz photoconductivity and carrier density. (b) NIR transient absorption kinetics (*λ*_pump_ = 603 nm, *λ*_probe_ = 970 nm, *I*_exc_ = 6.0 × 10^14^ photons cm^−2^ per pulse). Inset displays the corresponding photoluminescence spectra (*λ*_pump_ = 550 nm).

The early time THz kinetics of neat MAPbI_3_, MAPbI_3_ | Al_2_O_3_, and MAPbI_3_ | TiO_2_ are shown in Fig. 10(a). For neat MAPbI_3_ and MAPbI_3_ | Al_2_O_3_, a fast rise (instrument limited) of electron-hole pairs is followed by a second (∼2 ps) rise before reaching its maximum. Upon photoexcitation, electron-hole pairs are initially electrostatically bound and require an activation energy on the order of k*T* to dissociate into mobile charges. For MAPbI_3_ | TiO_2_, the THz transient rises faster than in the other two samples and grows with a very steep rise time. The response-limited rise in the THz photoconductivity for MAPbI_3_ | TiO_2_ was assigned to a sub-ps electron injection from the perovskite to TiO_2_. Moreover, the transient THz photoconductivity kinetics [Fig. 10(a), inset], normalized with excitation density, shows that the charge mobility of MAPbI_3_ | TiO_2_ is ∼7.5 cm^2^ V^−1 ^s^−1^, ∼3–4 times lower than in neat MAPbI_3_ and the MAPbI_3_ | Al_2_O_3_ (i.e., ∼20 cm^2^ V^−1 ^s^−1^), supporting the hypothesis that electron injection occurs in MAPbI_3_ | TiO_2_.

Optical TA kinetics of neat MAPbI_3_ and MAPbI_3_ | Al_2_O_3_ are shown in Fig. 10(b). It exhibits a fast, negative response followed by a ∼2 ps of further decrease. The time scale of this two component signals is consistent with the two-step rise in THz kinetics, confirming that charges are not instantaneously created. Furthermore, the negative signal is characteristic of stimulated emission, which agrees with the steady-state photoluminescence spectra [Fig. 10(b), inset], showing radiative recombination. In contrast, a single component ultrafast rise with positive sign occurs in the case of MAPbI_3_ | TiO_2_. This is consistent with the time scale of the appearance of electrons in TiO_2_ and identical to the rise time of the THz kinetics [Fig. 10(a)]. Although not complete, a strong quenching of the photoluminescence in MAPbI_3_ | TiO_2_ provides an additional evidence for electron injection.

**FIG. 11.**
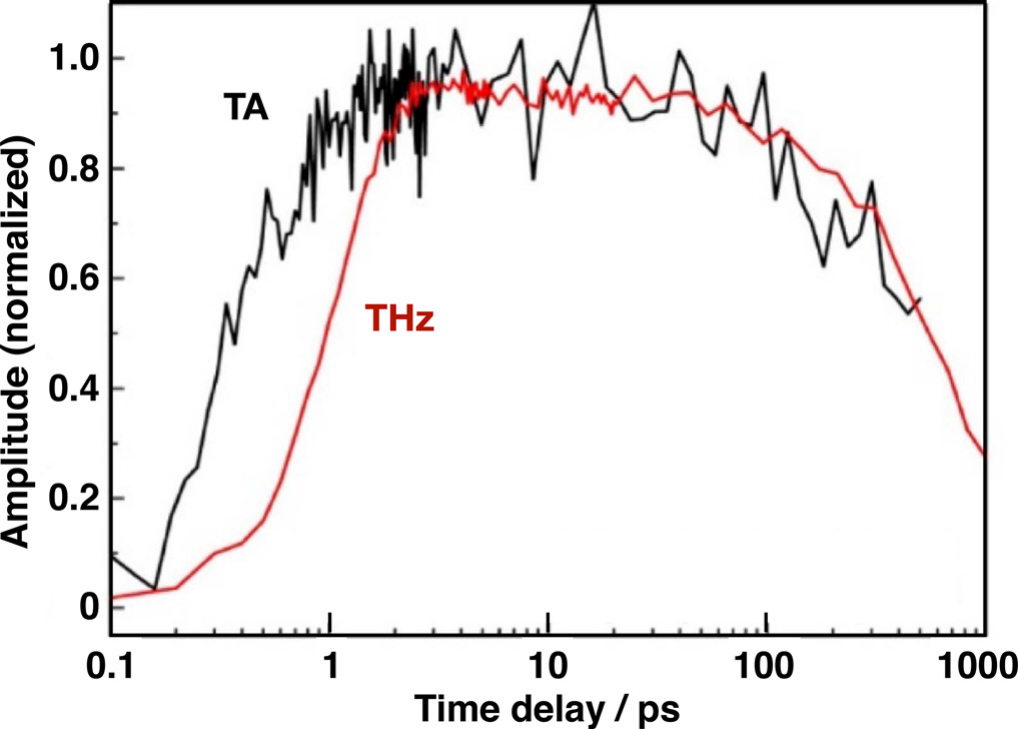
Comparison of NIR transient absorption (TA) and time-resolved terahertz (THz) kinetics for neat MAPbI_3_ showing that THz mobility remains constant for at least 1 nanosecond.

The THz photoconductivity kinetics on the tens of ps scale, for all three studied materials, exhibit a slow decay as shown in the inset of Fig. 10(a). Since the THz response is a product of carrier concentration and mobility, it is not possible to conclude, based on the THz kinetics alone, if this decay is due to charge recombination or to relaxation of the carriers. Optical TA, on the other hand, is a direct measure of the carrier concentration. In Fig. 11, kinetics of neat MAPbI_3_ were measured with TA and TRTS at similar intensities (∼10^13^ photons cm^−2^ per pulse). Within the experimental error, it was found that they are identical, implying that carrier mobility has to remain constant for at least 1 ns, otherwise a faster decay of the THz kinetics would be observed. Consequently, this means that the decay of the THz conductivity is a result of a decreasing carrier concentration. At the lowest excitation intensity (2.0 × 10^12^ photons cm^−2^ per pulse) where the charge carrier mobility is found to be highest, i.e., 25 cm^2^ V^−1 ^s^−1^, no decay is observed for the first 200 ps. This remarkably high THz mobility in solution-processed perovskite is consistent with the mobility recently obtained by Wehrenfennig *et al.*^107^

THz photoconductivity spectra were also measured 10 ps after photoexcitation. Within experimental error, both the amplitude and shape of the spectra of neat MAPbI_3_ and MAPbI_3_ | Al_2_O_3_ are identical. However, for MAPbI_3_ | TiO_2_, the spectral shape is qualitatively different, and the signal amplitude is approximately four times lower than for MAPbI_3_ and MAPbI_3_ | Al_2_O_3_ [as also shown in the THz signals of Fig. 10(a), inset].

From the above observations, a number of conclusions can be drawn: First, the identical THz spectra and photoconductivity kinetics of neat MAPbI_3_ and MAPbI_3_ | Al_2_O_3_ show that the presence of Al_2_O_3_ does not alter the dynamics and mobility of the charges in the perovskite, on the distance scale probed by the THz measurements (<100 nm). Second, the presence of TiO_2_ nanoparticles accelerates the formation of charge carriers, which leads to efficient electron injection in <1 ps, due to favorable band energy alignment of TiO_2_ and the perovskite. However, due to the low intrinsic mobility of conduction band electrons in TiO_2_, injection leads to unbalanced transport of charges, lowering the overall mobility.

Unbalanced electron and hole mobilities, which differ by orders of magnitude in bulk heterojunction solar cells, result in space charge-limited photocurrents lowering the power conversion efficiency.^108^ Therefore, it is important to assess the mobilities of both electrons and holes. From the THz measurements of porous TiO_2_films, it was shown that its intrinsic electron mobility is ≪1 cm^2^ V^−1 ^s^−1^. In this case, the electron mobility of TiO_2_ in MAPbI_3_ | TiO_2_ should be the same, i.e., the THz response is mainly due to the holes in the perovskite phase having a mobility of close to 7.5 cm^2^ V^−1 ^s^−1^. Consequently, from the measured THz mobility of ∼20 cm^2^ V^−1 ^s^−1^ for MAPbI_3_ and MAPbI_3_ | Al_2_O_3_, we can conclude that the electron mobility in the perovskite phase is ∼12.5 cm^2^ V^−1 ^s^−1^. The 12.5/7.5–2 ratio of electron and hole mobilities in the perovskite phase is in agreement with the recent theoretical calculations of the relative effective masses of electrons and holes.^109^ The finding that electron and hole mobility is almost balanced is a key information for the understanding of why pristine perovskite or perovskite | Al_2_O_3_ solar cells are so efficient.

Very slow microsecond time scale recombination at ambient solar intensities, assessed by additional time-resolved microwave conductivity measurements, in combination with high and almost equal electron and hole mobilities guarantees very efficient charge collection and thus high solar cell efficiency. The results also show that, as a consequence of electron injection from the perovskite to the TiO_2_ with very low electron mobility, the overall mobility is lowered. We note also that the lower Fermi level of TiO_2_ decreases the open circuit voltage leading to lower overall efficiency. A possible improvement of solar cell performance would be to engineer the active materials such that both electron and hole mobilities are on the level of the electron mobility in the perovskite.

Picosecond X-ray absorption spectroscopy was used to investigate the fate of charge carriers in Cs-based inorganic perovskites nanoparticles with atomic selectivity. A resolution of 80 ps was used and it was found that by then, hole forms small polarons at Br atoms, while electrons remain delocalized in the conduction band. Finally, no effect whatsoever was noted on the Cs atoms, in line with theoretical predictions.^110^

### C. Charge transport and transfer through D-A heterojunctions

**FIG. 12.**
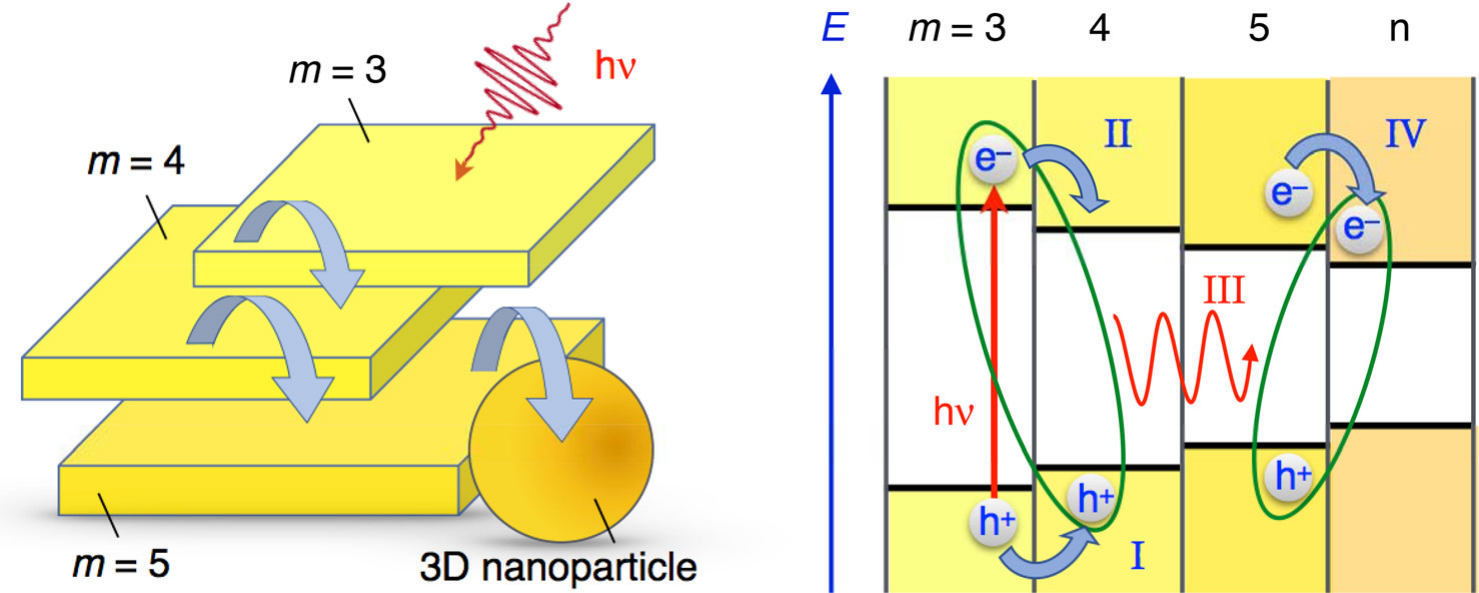
Cartoon illustrating the energy- and charge transfer processes occurring between the various nanostructures constituting MAPbBr_3_ perovskite colloidal aggregates. Left: Energy- and/or charge transfer cascade (curved blue arrows) between q-2D nanoplatelets of increasing thicknesses and eventually a 3D bulk-like nanoparticle. Right: Energetic scheme of some examples of photophysical processes taking place in a nanoparticle aggregate: Upon photoexcitation of a thin q-2D (m = 3) nanoplatelet, interfacial hole transfer can take place to the adjacent particle (process I). Electrostatic interaction of the hole with an electron remaining on the other side of the interface yields a CT exciton (green ellipse). Subsequent electron transfer (process II) leads to the excitation of the m = 4 q-2D nanoplatelet. Energy transfer to a neighbouring nanostructure characterized by a narrower bandgap is then possible (process III). Interfacial electron transfer (process IV) finally enables the formation of a new interfacial CT excitonic species.

Methylammonium lead tribromide MAPbBr_3_ perovskite nanoparticles suspensions in chlorobenzene contain various nanostructures: quasi-2D nanoplatelets of variable thickness and 3D bulk-like nanoparticles. These structures exhibit several optical signatures that were previously reported and assigned. The nanoplatelets are blue-shifted compared with the bulk perovskite, due to a significant confinement regime. Using a combination of steady-state, excitation-dependent ultrafast transient absorbance and time-correlated single photon counting (TCSPC) measurements, Bouduban *et al.* unraveled the presence of significant inter-structures interactions in the form of a cascade of energy and charge transfer, the latter being mediated by the formation of inter-particle charge transfer states.^111^ Upon photoexcitation, localized excitons are formed within one nanostructure. They either rapidly recombine, yielding a short-lived emission on the picosecond timescale, or turn into charge transfer excitons following the injection of one type of carrier into a narrower band-gap, neighboring nanostructure (Fig. 12). These charge transfer excitons possess a permanent dipole and submit the material at close proximity to an electric field, which produces a significant photoinduced electroabsorption contribution to the transient absorption spectra. Carrier pairs contained in CTE eventually recombine, resulting in the long-lived, microsecond emission observed by TCSPC.

Similar cascade charge transfer processes in highly efficient light-emitting diode (LEDs) and photovoltaic devices are likely to occur in materials characterized by a multigrain morphology. As much as long-distance radiative energy transfer within the active film of a perovskite solar cell (photon recycling), non-radiative energy transfer and inter-domain charge transfer mediated by interfacial CT states could play an important role in slowing down the recombination of photocarriers and increasing their diffusion length.

The time-resolved electroabsorption spectroscopy (TREAS) technique was successfully applied for the first time to a methylammonium lead triiodide perovskite multigrain film. The active material was prepared by vapor deposition and appeared to be polycrystalline with an average grain size of 40 nm. MAPbI_3_ subjected to an externally applied electric field on the order of 10 MV m^−1^ displayed a blue shift of its excitonic absorption edge at 780 nm, corresponding to a quadratic electroabsorption response compatible with both Stark and Franz-Keldysh-Aspnes models. The electroabsorption signal was exploited to probe optically the time evolution of the local electric field experienced by the perovskite.^112^

**FIG. 13.**
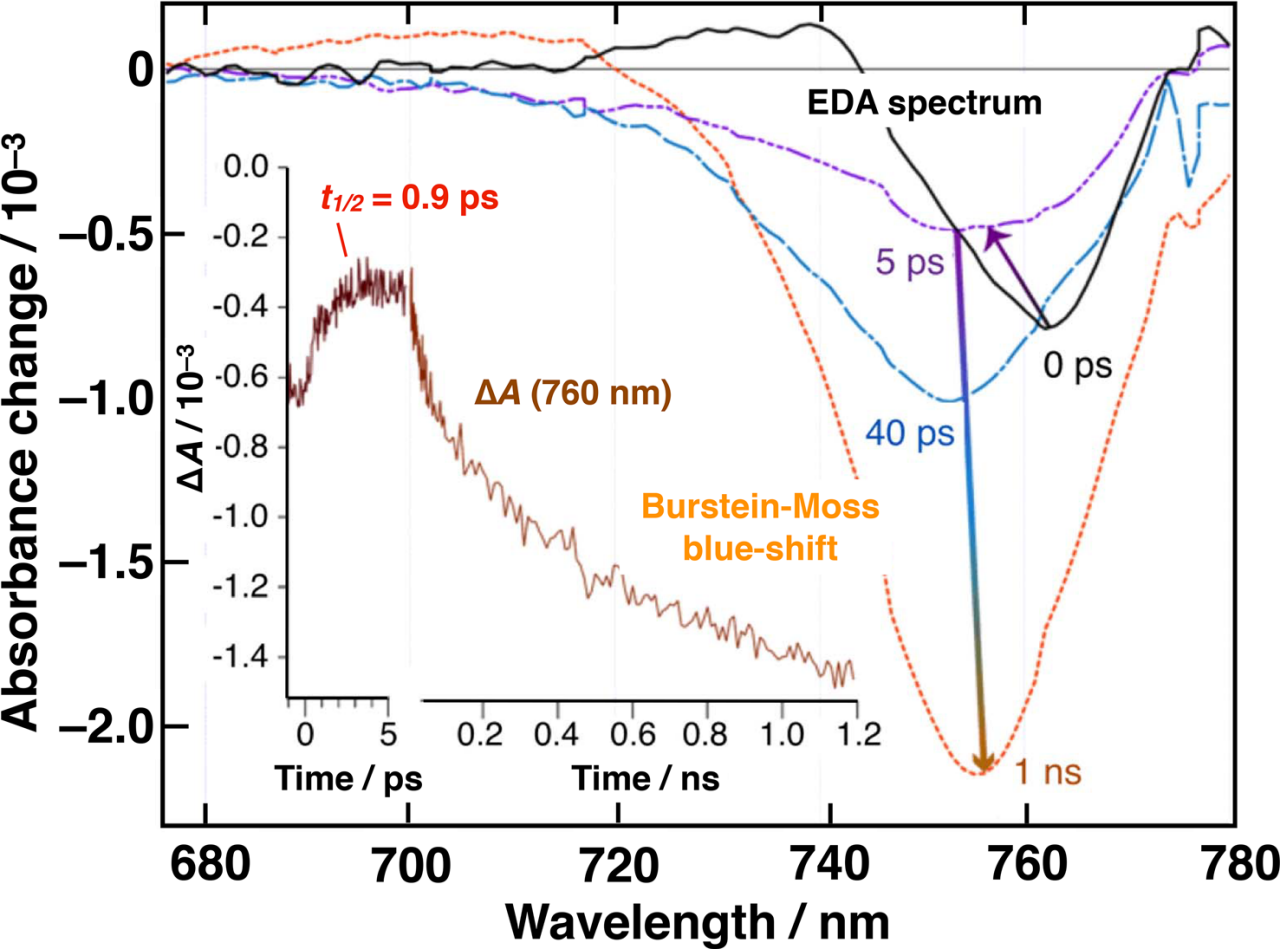
Time-evolution of electro-modulated differential absorption (EDA) spectra of insulated MAPbI_3_ films excited at *λ* = 545 nm and submitted to an external electric field *E*_0_ = 1.7 × 10^5^ V cm^−1^. Inset: Time-dependence of the differential absorbance change recorded in the same conditions at λ_probe_ = 762 nm.

Upon band-gap irradiation, electron–hole pairs were formed. Their initial spatial separation was observed from the differential electroabsorption signal dynamics to take place with a time constant of 0.94 ± 0.1 ps, until charges were trapped at grain boundaries (Fig. 13). An average intra-grain dc mobility of the carriers of *μ*_±_ = 23 ± 4 cm^2^ V^−1 ^s^−1^ was extracted from this result, in good agreement with terahertz spectroscopy measurements. A second charge separation step was observed optically with a time constant of 24 ± 4 ps. This kinetic component was assigned to the detrapping of carriers and their migration to the opposite insulated film surfaces, where they accumulated, producing a Burstein-Moss blue shift of the absorption spectrum of the MAPbI_3_ material. A value of the mobility, limited by trapping-detrapping processes at grain boundaries, of *μ*_n_ = 5.5 ± 1 cm^2^ V^−1 ^s^−1^ was estimated for electrons drifting across the entire film thickness. Importantly, charge recombination was observed to be entirely suppressed between field-separated carriers generated at initial densities of *n*_0_ ≤ 2 × 10^16^ cm^−3^.

The TREAS technique also proved quite powerful in characterizing the kinetics of the charge transmission between the perovskite absorber material and the carrier-extracting layers in fully operational photovoltaic devices. In particular, electron accumulation at the junction between the vapor-deposited MAPbI_3_ film and a mesoporous nanocrystalline TiO_2_ layer was observed before the charge extraction could take place at the subnanosecond time scale.^112^

**FIG. 14.**
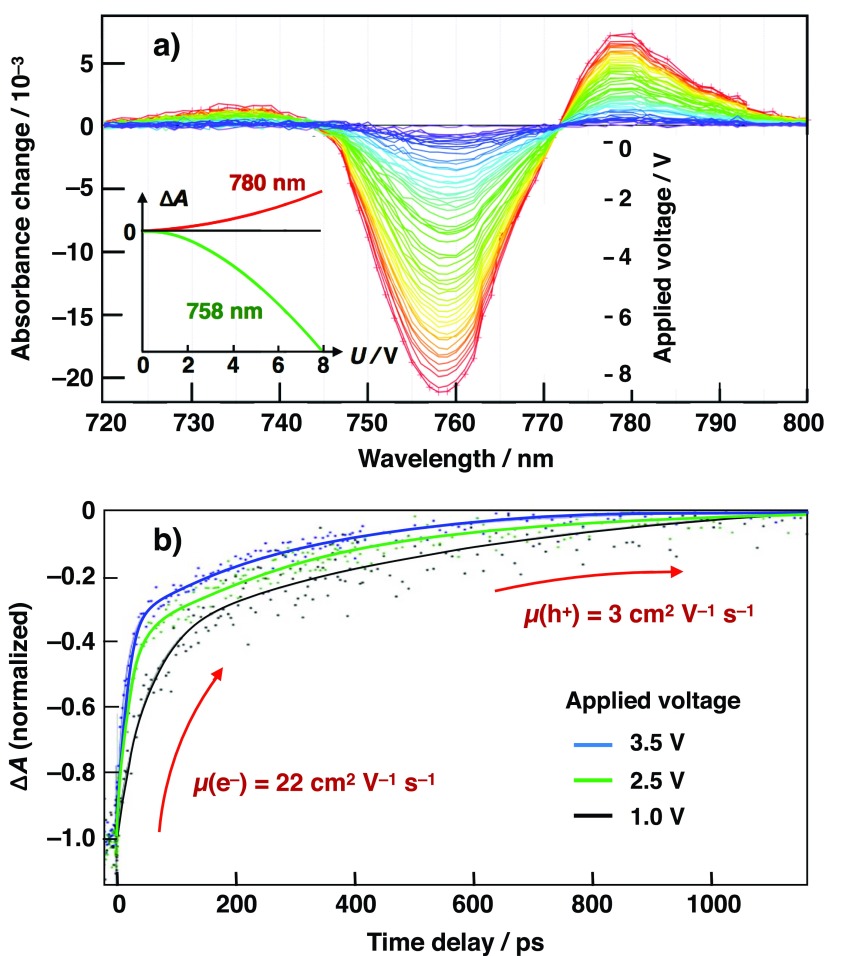
(a) Electro-absorption spectra of the perovskite film contained in a complete solar cell upon application of increasing voltages between both electrodes. Inset shows the quadratic dependence of the differential absorbance signal upon the applied external field intensity and direction. (b) Time-dependence of the EA differential absorbance change recorded upon application of an external electric field *E*_0_ = 4.9 × 10^4^ V cm^−1^ (*U*_0_* *= 3.5 V), *E*_0_ = 3.5 × 10^4^ V cm^−1^ (*U*_0_* *= 2.5 V), and *E*_0_ = 1.4 × 10^4^ V cm^−1^ (*U*_0_* *= 1.0 V) and probed at the peak of the electroabsorption signal at *λ* = 758 nm, energy fluence = 0.1 *μ*J cm^−2^.

Paraecattil *et al.* carried out ultrafast TREAS on mixed halide, mixed cation perovskite solar cells.^101,113^ These measurements present the first application of this technique to the investigation of complete functional perovskite solar cells. A quadratic electroabsorption response was observed in perovskite solar cells submitted to externally applied voltages (reverse bias) as low as 1 V [Fig. 14(a)]. It allowed the investigation of the electric field screening dynamics by photogenerated charge carriers with a monochromatic pump pulse at a low excitation fluence of 0.1 *μ*J cm^−2^ (carrier density 3 × 10^15^ cm^−3^). Experimental conditions resembled the electric field and optical excitation densities experienced by the device under operating conditions with sunlight. The screening dynamics of the electroabsorption signal were related to electron and hole drift to the perovskite | acceptor and perovskite | HTM interfaces, respectively [Fig. 14(b)]. The insights gained from measurements allowed to calculate carrier mobilities of 3 ± 1 cm^2^ V^−1 ^s^−1^ for holes and 21.9 ± *5* cm^2^ V^−1 ^s^−1^ for electrons in state-of-the-art perovskite solar cells. No significant difference in carrier dynamics was observed between substituting mesoporous TiO_2_ with SnO_2_ as the electron-accepting layer in devices. The TREAS technique measures electron and hole transport to the acceptor interface and not carrier injection from the perovskite into the acceptor. For both architectures, the observed signal was dominated by carrier transport across the bulk perovskite layer, which is comparable in the two devices. Transient absorption measurements on the perovskite devices revealed remarkably similar TA spectra at 500 fs after photo-excitation than with steady-state EA spectra of the same devices. Observations suggest that photoexcitation results in a transient photoinduced Stark shift of the perovskite ground state absorption spectrum, due to the electric field generated between electrons and holes and warrants further investigation.

**FIG. 15.**
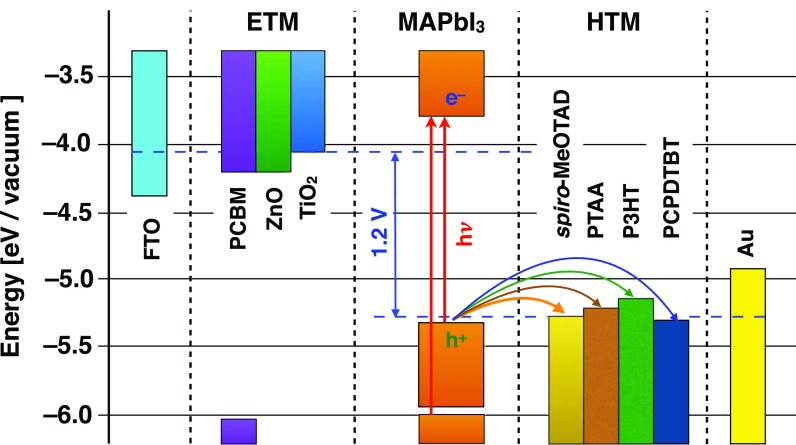
Simplified band alignment diagram for ETM | MAPbI_3_ | HTM double donor-acceptor heterojunctions. Curved arrows represent the hole transfer processes taking place from the valence band maximum of the perovskite absorber to the various investigated hole-transport materials.

**FIG. 16.**
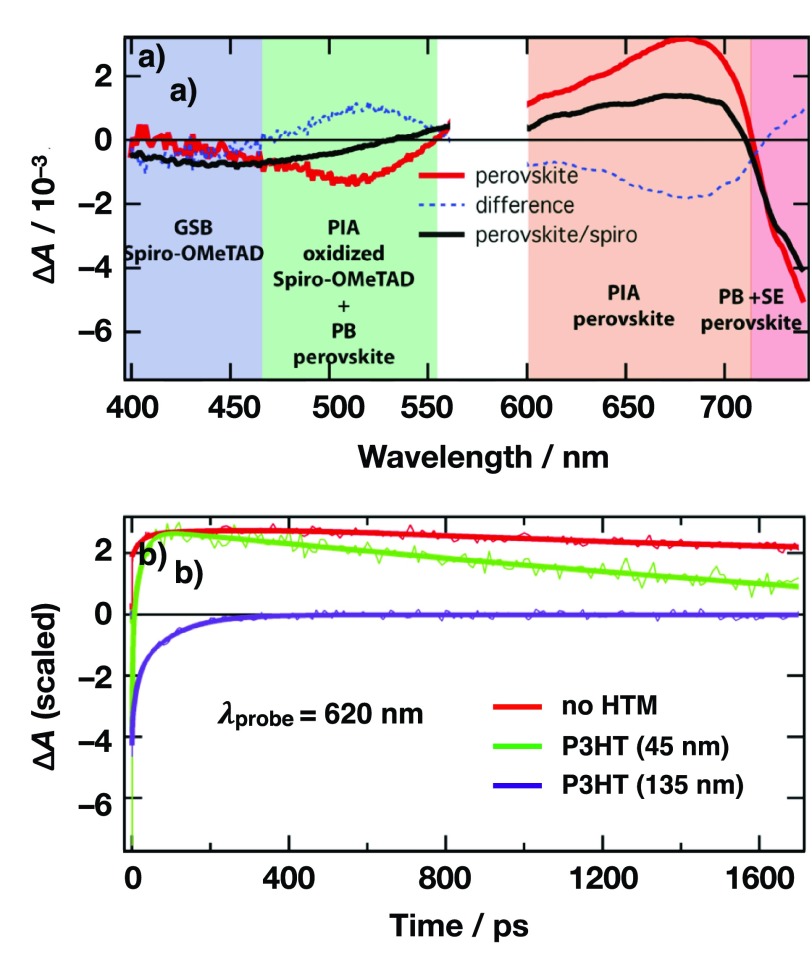
(a) Transient absorption spectra of mesoporous TiO_2_ | MAPbI_3_ and mesoporous-TiO_2_ | MAPbI_3_ | *spiro*-MeOTAD systems 1ps after excitation, as well as the corresponding difference spectrum. (b) Dynamics at 620 nm for mesoporous TiO_2_ | MAPbI_3_ and mesoporous TiO_2_ | MAPbI_3_ | P3HT.

The fundamental understanding of the working principle of perovskite-based solar cells requires understanding, on the one hand, the charge generation inside the perovskite as well as the charge transfer at the electron and hole extracting interfaces to optimize the device performance. Brauer *et al.* showed that upon band-gap resonant excitation, the formation of free charges within the MAPbI_3_ perovskite layer occurs via an excitonic state that dissociates into free charges in approximately 200 fs.^114^ This fast dissociation is consistent with previously published exciton binding energies of a few meV. When excess energy is provided during excitation, free charges are formed directly and cool down to the band edge in a few hundreds of picoseconds while the excitonic signature is not obeserved.^114^ Following the excitation of perovskite, the hole transfer from photo-excited MAPbI_3_ to *spiro*-MeOTAD was investigated. It has been shown that the hole transfer is essentially ultrafast occurring on a sub-80 fs time scale.^114^ This timescale is faster than carrier thermalization and extraction of hot holes has been observed. Changing from the small molecular hole transporter *spiro*-MeOTAD to polymeric hole transporter like PTAA, P3HT, and PCPDTBT (Fig. 15) hole injection from the photo-excited perovskite is slowed down and the hole transfer occurs on a time scale of a few nano-seconds from thermalized states and is independent of excess excitation energy (Fig. 16).^115^ Consequently, it has been shown that energy transfer from the photoexcited P3HT to MAPbI_3_ can occur, provided the excitation in the P3HT is in close proximity to the P3HT | MAPbI_3_ interface. In this way, P3HT can be used to sensitize MAPbI_3_ and excitations in the HTM are not lost for photocurrent generation.^115^ The differences in hole transfer rates between small molecular HTMs as *spiro*-MeOTAD and polymeric HTMs as P3HT might be partially explained by poor physical contact in the case of the polymers, given that the thermodynamic driving force for hole transfer to all investigated HTMs is rather similar. Furthermore, the ultrafast hole transfer in *spiro*-MeOTAD suggests an extraordinarily good coupling of its LUMO to the perovskite valence band.

## VI. PERSPECTIVE AND FUTURE DIRECTIONS

Several ultrafast spectroscopy techniques have been developed with the aim of providing the necessary tools to scrutinize charge carrier dynamics at donor-acceptor heterojunctions that are central to third-generation photovoltaic and LED devices. Results obtained so far have been quite successful but call for a deeper and more fundamental investigation of the dynamics of free- and bound photo-generated carriers, particularly in OPV and lead halide perovskite materials.

One of the outstanding issues regarding the charge carrier dynamics in photovoltaic materials is the clear identification of the fundamental excitations at and below the band gap.^37^ The foreseen approach by the Chergui group to address them will be to combine resonant inelastic X-ray scattering (RIXS) studies with angle-resolved photoelectron spectroscopy (ARPES) and ultrafast 2D UV spectroscopy. This unique combination of tools will also be used to investigate the charge carrier dynamics, in particular that of holes, which have so far escaped observation. As far as perovskites are concerned,^110^ of great interest is the understanding of spin-orbit coupling and the critical role of local inversion-symmetry breaking fields, which leads to phenomena such as Rashba splitting and possible spin polarization for future applications. Understanding the interaction of molecules with the surface of materials is another subject of crucial importance for energy-related applications. Time-resolved 2D UV and surface-selective ultrafast ARPES will allow for probing adsorbates at the TiO_2_ (110) surface and perform orbital tomography on their LUMOs, which represents the doorway to either electron injection or photocatalytic processes.

The exact mechanism by which electrons and holes overcome trapping and fast recombination to yield free carriers in materials at the base of OPV and perovskite devices is still debated.^14^ Increasing evidence points to the critical role of large polarons and hot charge transfer excitons in assisting this process. The precise properties of incoherent excitonic and polaronic species, as well as trapped carrier populations will be the focus of future research efforts of the Moser group.

Conventional experimental techniques, such as ultrafast transient absorption and broadband fluorescence up-conversion, will be supplemented by direct quasi-particle probing using a combination of time-resolved electroabsorption and ultra-broadband time-resolved terahertz spectroscopies. TREAS will be used to investigate the electron and hole drift mobilities in organic photovoltaic devices and perovskite solar cells and provide the necessary insight into charge trapping and detrapping processes and the possible implication of CTEs at grain boundaries.^51,112^ TRTS will allow to distinguish between free carriers and excitonic species involved in the various recombination pathways. This technique will also enable the identification of phonons involved in indirect transitions and in the formation of polarons through the direct monitoring of the time-evolution of their resonant bands.

Pump-push-probe and pump-dump-probe spectroscopy techniques have been used to investigate the properties of excitons and charge-transfer states of conjugated polymers.^116–118^ A real-time view of bound charge states formation and relaxation will be provided for dye-sensitized, small molecule-based OPV, and perovskite solar cells systems using a comparable approach. A time-resolved optical pump-IR push-terahertz probe spectroscopy (PPTPS) scheme will be employed to scrutinize excitons, CTE, polaron, and trapped carrier dynamics in various systems and conditions and monitor directly the time evolution of their binding energy.

Time-resolved terahertz spectroscopy provides key insights into quantum confinement phenomena and carrier transport in nanomaterials. Conventional THz spectroscopy, however, is restricted by the diffraction limit to measurements of the dielectric functions averaged over the size, structure, orientation, and density of nanoparticles, crystal grains, or nanodomains constituting the material under study. Ultrafast THz nanoscopy combines femtosecond optical excitation with sub-cycle broadband terahertz probing and scattering-type near-field scanning optical microscopy (s-NSOM). This technique was recently shown to allow for imaging the complex dielectric function of nanostructured materials in three dimensions (x, y, t) with 10 nm spatial- and sub-100 fs time-resolution.^119^ A similar experimental approach will enable the characterization of carrier dynamics in OPV systems and in hybrid perovskite thin film solar cells and quantum-confined nanostructures for LED and laser applications. Important scientific challenges will be to map the carrier density across the active film thickness and at heterojunctions, to determine local doping and space charge layers, to image the spatial distribution of trap states and recombination centers and to monitor the ultrafast time evolution of these various properties.

## NOMENCLATURE

AFM: Atomic-force microscopy
AM1.5: Solar irradiance at mid-latitudes, where the sunlight has to pass through 1.5 atmosphere thickness, corresponding to a solar zenith angle *z* = 48.2°
ARPES: Angle-resolved photoelectron spectroscopy
BHJ: Bulk heterojunction
CC2: Second-order approximate coupled cluster computational method
CT(S/E): Charge transfer (state/exciton)
DAH: Donor-acceptor heterojunction
D-B-A: Bridged donor-acceptor molecule
DSSCs: Dye-sensitized solar cells
D-π-A: Conjugated π-bridged donor-acceptor dye molecule
EA: Electro-absorption
EDA: Electro-modulated differential absorption spectroscopy
ET: Electron transfer process
ETM: Electron-transport material
FA: Formamidinium (= CH_5_N_2_)
HOMO: Highest occupied molecular orbital
HTM: Hole-transport material
LED: Light-emitting diode
LUMO: Lowest unoccupied molecular orbital
MAPbI_3_: Methylammonium lead triiodide (= CH_3_NH_3_PbI_3_)
NSOM: Near-field scanning optical microscopy
OPV: Organic photovoltaics
PBDTTPD: Poly[[5-(2-ethylhexyl)-5,6-dihydro-4,6-dioxo-4H-thieno[3,4-c]pyrrole-1,3-diyl][4,8-bis[(2-ethylhexyl)oxy]benzo[1,2-b:4,5-b′]dithiophene-2,6-diyl]]
PBTTT: Poly[2,5-bis(3-tetradecylthiophen-2-yl)thieno[3,2-b]thiophene]
PCBM: Phenyl-C_61_-butyric acid methyl ester (soluble derivative of fullerene)
PCPDTBT: Poly[2,6-(4,4-bis-(2-ethylhexyl)-4H-cyclopenta [2,1-b;3,4-b′]dithiophene)-alt-4,7(2,1,3-benzothiadiazole)] conjugated polymer semiconductor
PL: Photoluminescence (= fluorescence, in the case of an allowed transition)
PPTPS: Time-resolved optical pump-IR push-terahertz probe spectroscopy
PSCs: Perovskite solar cells
PTAA: Poly[bis(4-phenyl)(2,4,6-trimethylphenyl)amine] polymer semiconductor
P3HT: Poly(3-hexylthiophene-2,5-diyl) conjugated polymer semiconductor
RIXS: Resonant inelastic X-ray scattering
*spiro*-MeOTAD: N^2^,N^2^,N^2′^,N^2′^,N^7^,N^7^,N^7′^,N^7′^-octakis(4-methoxyphenyl)-9,9′-spirobi[9H-fluorene]-2,2′,7,7′-tetramine (= *spiro*-OMeTAD)
TA(S): Transient absorption (spectroscopy)
TCSPC: Time-correlated single-photon counting (PL lifetime measurement)
(TD)DFT: (Time-dependent) density functional theory
TREAS: Time-resolved electro-absorption spectroscopy
TRMC: Time-resolved microwave conductivity
TRTS: Time-resolved terahertz spectroscopy
